# Randomized crossover clinical studies to assess abuse liability and nicotine pharmacokinetics of Velo Oral Nicotine pouches

**DOI:** 10.3389/fphar.2025.1547073

**Published:** 2025-03-13

**Authors:** Milly N. Kanobe, Christie Y. Powell, Makena Patrudu, Sarah A. Baxter, Melissa A. Tapia, John Darnell, Kristen Prevette, Alison G. Gibson, Sarah A. Ayoku, Leanne Campbell, Jeffrey W. Coffield, Brian M. Keyser, Bhagya Sukka Ganesh, Nathan Gale, Kristen G. Jordan

**Affiliations:** ^1^ RAI Services Company, Winston-Salem, NC, United States; ^2^ BAT (Investments) Limited, Research and Development, Southampton, United Kingdom

**Keywords:** nicotine uptake, abuse liability, subjective effects, Velo Oral Nicotine pouches, combustible cigarettes, NRT gum, tobacco harm reduction

## Abstract

**Introduction:**

Oral nicotine pouches (ONPs) are a newer category of smokeless tobacco products containing pharmaceutical-grade nicotine but no tobacco leaf. These products have the potential to help smokers transition away from cigarettes. To assess their potential role as alternatives to cigarettes, we evaluated the abuse liability (AL) of Velo ONPs with varying nicotine content (4–12 mg per pouch), pouch size (600 mg or 400 mg) and flavor (six varieties) in comparison to high (cigarettes) and low (nicotine replacement therapy [NRT] gum) AL comparators.

**Methods:**

Independent randomized crossover clinical studies were conducted to assess AL, including subjective effects (product liking [PL], urge to smoke, product effects, overall PL, and overall intent to use again) and nicotine pharmacokinetic (PK) parameters of Velo ONPs. Participants used test products under controlled conditions, and subjective effect measures were collected using validated questionnaires. Nicotine PK parameters, including peak nicotine concentration (C_max_), time to maximum concentration (T_max_), were assessed.

**Results:**

Mean PL scores for all Velo ONPs (p < 0.0042) and Velo Mini Pouches (p < 0.0031) were significantly lower than cigarettes, regardless of nicotine level, pouch size, or flavor, but similar to NRT gum. Other subjective measures for Velo ONPs were less favorable than cigarettes and comparable to or lower than NRT gum. Nicotine uptake with Velo ONPs was slower (reflected by a longer T_max_) and had lower C_max_ than cigarettes but was comparable or slightly lower than NRT gum. Overall nicotine uptake increased with increasing nicotine content and was comparable to that of cigarettes for Velo ONPs with higher nicotine levels. Flavor had no effect on nicotine uptake of Velo ONPs.

**Discussion:**

Velo ONPs demonstrated an AL profile lower than cigarettes and similar to NRT gum, suggesting a reduced potential for abuse compared to cigarettes. The slower nicotine uptake and lower peak nicotine levels further support their potential as a lower-risk alternative. These findings highlight the potential role of ONPs in tobacco harm reduction strategies by providing an alternative nicotine source with a lower AL than combustible cigarettes.

**Systematic Review Registration:**

The clinical studies were registered at ClinicalTrials.gov; NCT05129657, NCT05294497, and NCT05081154.

## Introduction

The current tobacco product marketplace is highly diverse and has been evolving over the past decade with the emergence of novel tobacco and nicotine products including electronic cigarettes, heated tobacco products, and oral nicotine pouches (ONPs) (University of Bath, 2023). A continuum of risk has been recognized among tobacco and nicotine products that deems combustible products (e.g., cigarettes) as having the highest health risk and medicinal nicotine products as conveying the lowest risk (Gottlieb and Zeller, 2017; King et al., 2023). Smokeless tobacco products (STPs) are associated with significantly lower risks to health than cigarettes and are closer to medicinal nicotine products along the risk continuum (Abrams et al., 2018; Institute of Medicine, 2001). In particular, use of oral STPs, such as moist snuff and snus, has been associated with lower disease risk relative to cigarette smoking (Henley et al., 2007; Henley et al., 2005; Luo et al., 2007), primarily because oral STP users have a significantly reduced exposure (Clarke et al., 2019; Grandolfo et al., 2024) to the numerous harmful and potentially harmful constituents (HPHCs) (Food and Drug Admini, 2012) formed during tobacco combustion, which the smoker inhales, and have been identified as causative agents of several serious diseases (Centers for Disease Control and Prevention, 2023).

Tobacco harm reduction (THR) is a public health risk mitigation strategy aimed at reducing the health burden associated with cigarette smoking (Hatsukami and Carroll, 2020). The fundamental principle of THR is to encourage smokers who are unable or unwilling to stop smoking to switch to use of tobacco and/or nicotine-containing products that deliver nicotine but with reduced exposure to HPHCs and other toxicants, and have a positive impact on both individual and population-level health (Institute of Medicine, 2001). In the United States, the Food and Drug Administration (FDA) has indicated they “*will focus on a regulatory framework that focuses on and supports innovation to promote harm reduction*” (Gottlieb and Zeller, 2017). The FDA has authorized that some snus and moist snuff products can be marketed as “modified risk tobacco products” (Food and Drug Administration, 2019; Food and Drug Administration, 2023), based partly on the reduced exposure profile of these products relative to cigarettes. While these oral STPs do not generate or elicit exposure to combustion-related toxicants (i.e., the main drivers of smoking-related diseases), they do contain residual levels of some HPHCs, such as tobacco-specific nitrosamines (Back et al., 2023; Borgerding et al., 2012).

Oral nicotine pouches (ONPs) are an emerging category of nicotine products that contain pharmaceutical-grade nicotine, but no tobacco leaf (Grandolfo et al., 2024; Jackson et al., 2023; Shaikh et al., 2023). They are typically portioned powder mixtures comprised of nicotine, flavorings, and other ingredients contained in a porous material termed “fleece”. During use, the pouches are placed between the upper lip and gum, where nicotine is absorbed into the bloodstream through the buccal mucosa. Although ONPs are a relatively recent product innovation, growing evidence indicates that they may be a reduced exposure product that conveys lower health risks compared to both cigarettes and oral STPs, such as moist snuff and snus. For example, levels of some HPHCs are significantly lower in ONPs than in snus (Back et al., 2023; Azzopardi et al., 2022a; Jablonski et al., 2022). In addition, a clinical study found lower levels of biomarkers of exposure and improved levels of biomarkers of potential harm among ONP users compared with cigarette smokers (Azzopardi et al., 2023). Furthermore, biomarkers of both exposure and potential harm among ONP users were at levels similar to former and never smokers (Azzopardi et al., 2023).

Combustible cigarettes exhibit a high degree of abuse liability (AL), a term synonymous with dependence potential (Carter et al., 2009a; Vansickel et al., 2021; Maloney et al., 2019). This high AL contributes both to increased physical and behavioral dependence on cigarettes and difficulty in stopping smoking. Given this, the AL of tobacco products is a critical attribute in determining the likelihood of both continued use and use frequency, factors that impact the degree of exposure to the combustion- and tobacco-derived toxicants that cause adverse health effects and increase harm (Carter et al., 2009b). In terms of THR and the ability of novel tobacco and nicotine products to reduce harm among smokers, possessing at least some degree of AL is important, as the new product should be a suitable substitute for cigarettes or support switching away from smoking (Abrams et al., 2018; Ashley et al., 2020; Cahn et al., 2021; Fearon et al., 2022). Conversely, high AL or a high dependence potential might lead to the novel product posing an initiation or addiction risk to non-users of nicotine products, particularly among susceptible populations such as youth and young adults (Ashley et al., 2020; Cahn et al., 2021). Thus, assessment of AL is an important component of determining whether the introduction of a novel tobacco product into a market is appropriate for the protection of public health in terms of its impact on the population as a whole (Congress, 2009).

The AL of a novel nicotine-containing tobacco product is a composite measure based predominantly on the pharmacokinetic (PK) and subjective effects associated with the use of that product (Fearon et al., 2022; Henningfield and Keenan, 1993; Vansickel et al., 2022). Despite the growing popularity of ONPs (Majmundar A et al., 2022), only a limited number of studies have evaluated these factors to assess their AL. In three separate clinical studies, we evaluated nicotine uptake and subjective effects of a range of Velo ONPs varying in nicotine content, pouch size, and flavor in order to provide an overall assessment of the AL of these products and to determine whether they may contribute both to THR and to improving overall population-level health.

## Materials and methods

### Study design

In three separate randomized, open-label, crossover, in-clinic confinement studies, subjective effects and nicotine uptake of Velo ONPs were evaluated among adult smokers. The studies were registered at Clinicaltrials.gov (NCT05129657, NCT05294497, NCT05081154) and conducted between October 2021 and June 2022 in the United States (US). They were approved by the Advarra Institutional Review Board (Columbia, MD, United States) and were conducted in accordance with the Declaration of Helsinki and applicable sections of the US Code of Federal Regulations and ICH E6 Good Clinical Practice. All participants provided written informed consent to participate.

### Study participants

The study populations consisted of adult smokers of filtered, non-menthol or menthol cigarettes who self-reported daily smoking of at least 10 cigarettes for at least 6 months. Potential participants were identified from a database of healthy volunteers held at the study site, and/or through advertisements on radio/social media directed at the target population. The main inclusion criteria were age 21–60 years, general good health, and a response of less than 30 min for time to first cigarette use in a day on the Fagerström test for nicotine dependence (FTND) (Fagerström and Eissenberg, 2012; Heatherton et al., 1991). The main exclusion criteria were pregnancy and breastfeeding. Cigarette smoking was confirmed at screening by measuring exhaled carbon monoxide (ECO) and urine cotinine. Eligibility criteria required an ECO between ≥10 ppm and ≤100 ppm, along with a positive urine cotinine test using a dipstick at a 200 ng/mL cutoff concentration. Smokers who also used smokeless tobacco products (e.g., snus, moist snuff) were eligible to participate in the study. Attempts were made to recruit at least 15%–20% African American participants as representative of the US population of smokers (Jamal et al., 2016).

### Study products

The study products were Velo ONPs (American Snuff Company, LLC, Winston-Salem, NC, United States): portioned oral nicotine products manufactured using tobacco-derived, pharmaceutical-grade nicotine, flavorings, and other ingredients specific to the flavor concentrate of the product. In Study 1 (Velo Pouch AL), three Velo Pouches [600 mg each] in one flavor (Cool Mint), with different nicotine levels (4, 8, and 12 mg nicotine) were evaluated; in Study 2 (Velo Mini Pouch AL), four Velo Mini Pouches [400 mg each] in two flavors (Cool Mint and Modern Traditions) and two nicotine levels (4 and 8 mg nicotine) were evaluated; and in Study 3 (Velo Pouch PK), seven Velo Pouches [600 mg each] in six flavors (Cool Mint, Modern Traditions, Berry Frost, Cinnamon, Wintergreen, and Smooth) and two nicotine levels (8 and 10 mg nicotine) were evaluated (Table 1). In Studies 1 and 2, the participant’s usual brand (UB) combustible cigarette and a commercially available NRT gum (Nicorette White Ice Mint polacrilex gum, 4 mg nicotine; Glaxo SmithKline, Durham, NC, United States) were included as high- and low-AL comparator products, respectively.

**TABLE 1:** Study products.

Product	Nicotine content (mg)	Flavor	Flavor category
*Study 1: Velo Pouch AL*			
Velo Pouch 600 mg	4	Cool Mint	Menthol Mint
Velo Pouch 600 mg	8	Cool Mint	Menthol Mint
Velo Pouch 600 mg	12	Cool Mint	Menthol Mint
UB Cigarette^a^	NA	NA	NA
NRT gum^b^	4	White Ice Mint	Nicorette gum
*Study 2: Velo Mini Pouch AL*			
Velo Mini Pouch 400 mg	4	Cool Mint	Menthol Mint
Velo Mini Pouch 400 mg	8	Cool Mint	Menthol Mint
Velo Mini Pouch 400 mg	4	Modern Traditions	Tobacco
Velo Mini Pouch 400 mg	8	Modern Traditions	Tobacco
UB Cigarette^a^	NA	NA	NA
NRT gum^b^	4	NA	NA
*Study 3: Velo Pouch PK*			
Velo Pouch 600 mg	10	Modern Traditions	Tobacco
Velo Pouch 600 mg	8	Modern Traditions	Tobacco
Velo Pouch 600 mg	8	Cool Mint	Menthol Mint
Velo Pouch 600 mg	8	Berry Frost	Fruit
Velo Pouch 600 mg	8	Cinnamon	Spice
Velo Pouch 600 mg	8	Wintergreen	Mint
Velo Pouch 600 mg	8	Smooth	Tobacco

^a^ Participants’ usual brand (UB) of combustible cigarette with either menthol or non-menthol flavor.

^b^ Nicorette White Ice Mint polacrilex gum, 4 mg nicotine.

Abbreviations: AL, abuse liability; mg, milligram (of nicotine); NA, not applicable; NRT, nicotine replacement therapy; PK, pharmacokinetic.

### Study protocols

**FIGURE 1:**
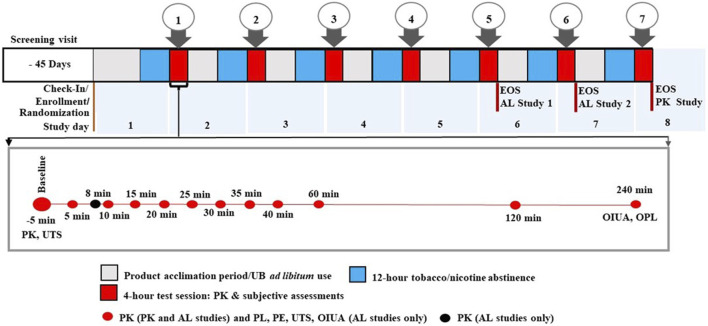
Summary of overall study design for Velo ONP studies. The Figure includes detailed overview of the timing of nicotine pharmacokinetics (PK) blood draws and completion of subjective effects questionnaires within each 4-h test session. Pharmacokinetics measurements were taken at the indicated timepoints in all three studies, except that Study 3 did not include an 8-min timepoint; product liking (PL), product effects (PE), and urge to smoke (UTS), and overall intent to use again (OIUA) were only administered in Studies 1 and 2; UTS was the only subjective measure administered at baseline in Studies 1 and 2 only; Overall product liking (OPL) was the only subjective measure administered in the Study 3. Both OPL and OIUA were administered at only the 240-min time point. Abbreviations: AL, abuse liability; EOS, end of study; min, minute; UB, usual brand; Velo ONP, Velo oral nicotine pouches.

The three in-clinic confinement studies lasted 6–8 days and followed similar protocols (Figure 1). On Day 1, participants who met all inclusion criteria and none of the exclusion criteria were enrolled, randomized to a product use sequence (assigned by the contract research organization (CRO) statistician) based on a Williams Design, and confined at the study site. Participants in Studies 1 and 2 brought with them a sufficient supply of their UB cigarettes to last through the confinement period. On Day 1, participants took part in a half-day pre-test-session to familiarize themselves with using the Velo ONPs and NRT gum (AL studies only). During the pre-test-session, they also had access to their UB cigarettes for *ad libitum* use. After this session, participants abstained from the use of all tobacco- and nicotine-containing products for a minimum of 12 h prior to each test session, and from any caffeine-containing products for 4 h, prior to and through the end of each test session.

Starting on the morning of Day 2, product use sessions were carried out daily based on the randomization sequence. After baseline measurements, participants in all studies used their designated Velo ONPs for approximately 30 min (additionally, those in the AL studies, either used one piece of NRT gum [per package labeling] for approximately 30 min or smoked one UB cigarette for up to 5 min). During the 240-min test session, blood samples for plasma nicotine PK parameters were collected and subjective effects questionnaires were completed. Following completion of each day’s test session, the participants were instructed to continue with product familiarization using their assigned product for the following day’s test session. Participants were also permitted to smoke their UB cigarettes during this period until the tobacco- and nicotine-containing product abstinence period (at least 12 h prior to each test session). The study procedures and timelines, including the points of nicotine uptake and subjective effects assessments, are depicted in Figure 1.

### Study endpoint measures

In Studies 1 and 2, AL was assessed as per established methods (Carter et al., 2009b; Fearon et al., 2022; Henningfield and Keenan, 1993; Vansickel et al., 2022) and regulatory guidance (Food and Drug Administration, 2021), and included measurements of nicotine pharmacokinetic and five subjective effects measures. Study 3 included measurements of nicotine pharmacokinetics and one subjective effects measure.

#### Nicotine pharmacokinetics

Nicotine pharmacokinetic parameters in all studies were determined as described previously (Campbell et al., 2023; Campbell et al., 2022; Stiles et al., 2017; Stiles et al., 2018). Blood samples were collected during product use sessions at specific timepoints from 5 min before (baseline) to 240 min after the start of product use (*see* Figure 1). Individual nicotine concentrations were baseline-adjusted using a model assuming that nicotine elimination follows first-order kinetics (Benowitz et al., 2006; Shiffman et al., 2009). The endpoints were maximum baseline-adjusted plasma nicotine concentration (C_max_), area under the curve (AUC) for 0–15 min (AUC_0-15_), AUC for 0–240 min (AUC_0-240_), and time to reach C_max_ (T_max_). Given the consistent presence of extreme T_max_ values across multiple studies, we have adopted the standard practice of using nonparametric methods, such as reporting medians for T_max_, to provide a more robust and accurate representation of the data.

#### Subjective effects

The AL studies evaluated five subjective effects measures through responses to questionnaires administered at various timepoints during the test sessions; the urge to smoke (UTS) questionnaire was also administered 5 min before product use (*see* Figure 1). Each questionnaire asked a single question with responses on a 10-point numerical rating scale (0, “Not at all”; 10, “Very much”) (Campbell et al., 2022; Stiles et al., 2017; Stiles et al., 2018). The subjective measurements included product liking (PL; “At this moment, how much do you like the product?“); UTS (“How strong is your current urge to smoke your usual brand cigarette?“); positive product effects (PE; “Rate the degree to which you feel positive effects of the product at this moment”) and negative PE (“Rate the degree to which you feel negative effects of the product at this moment”). Additionally, overall intent to use again (OIUA; “Rate the degree to which you would like to use the product”) and overall product liking (OPL; “Overall, how much did you like the product?“) were assessed at the 240-min timepoint.

The primary endpoints for the AL studies were (1) area-under-the-effect curve (AUEC) for PL for 5–240 min (AUEC_PL5-240_) and (2) the maximum PL effect (E_max PL_) after the start of product use. Secondary endpoints included AUEC for positive PE for 5–240 min (AUEC_PEpos 5–240_), maximum positive PE (E_max PEpos_), AUEC for negative PE for 5–240 min (AUEC_PEneg 5–240_), maximum negative PE (E_max PEneg_), minimum UTS (E_min UTS_), AUEC for UTS for 0–15 min (AUEC_UTS 0–15_), AUEC for UTS for 0–240 min (AUEC_UTS 0–240_), and effect of OPL (E_overall PL_) at 240 min. The only subjective assessment in the PK study was E_overall PL_ (*see* Figure 1).

### Safety and adverse events assessments

Participant safety was monitored throughout the study by assessments of adverse events (AEs), clinical laboratory tests, and vital signs measurements, as well as by physical or oral examinations. Symptom-driven physical examinations were performed as needed at the discretion of the Principal Investigator. An AE was coded by primary system organ class and preferred term according to the Medical Dictionary for Regulatory Activities version 24.1 (MedDRA Maintenance and Support Services Organization, 2021). The severity of an AE was categorized as mild, moderate, or severe; in addition, the relationship of the AE to study product use (not related, unlikely related, possibly related, or related) as determined by the Principal Investigator, was recorded.

## Data analysis

### Sample size calculation

For Studies 1 and 2, sample sizes were determined to achieve 90% power to detect the hypothesized difference of 2.2 (σ = 2.07) in the maximum PL score between the Velo ONP and UB cigarette, using a two-sided test for differences with Bonferroni-adjusted α levels of 0.0042 (Study 1) and 0.0031 (Study 2). This hypothesized difference, the smallest detectable among all the primary endpoint comparisons, served as the basis for sample size calculation. As a result, a minimum of 40 and 36 participants were required for Studies 1 and 2, respectively. To account for attrition and maintain a balanced Williams design, 50 participants were randomized for Study 1 and 43 for Study 2.

For Study 3, sample size determination was based on the goal of achieving a 95% confidence interval with a half-width that is within 20% of the primary endpoint mean values. The target number of randomized participants for this study was a minimum of 42 subjects, allowing for approximately a 33% dropout rate, with a goal of having at least 28 participants completing the study.

### Analysis populations

All participants who completed at least one post-baseline/study product use session were included in the statistical analysis of subjective effects unless their subjective effects profiles were deemed unevaluable. All randomized participants who used at least one study product were included in the nicotine pharmacokinetic parameter analyses. Each individual pharmacokinetic profile was examined for completeness and only data from participants with an evaluable pharmacokinetic profile were included in the analysis of nicotine uptake parameters. The safety population comprised all enrolled participants.

### Statistical analysis in studies 1 and 2

For analysis of the primary subjective effects assessments, AUEC_PL 5–240_ and E_max PL_ for each Velo ONP were compared to those for both the high- and low-AL comparators (UB cigarette and NRT gum, respectively) by using a mixed effects model analysis of variance (ANOVA) with Bonferroni correction for multiple comparisons using an alpha level of 0.0042 in Study 1 (12 comparisons) and 0.0031 in Study 2 (16 comparisons). The mixed effects ANOVA model was specified as follows:

$$y_{ijkp}=µ+\alpha_{i}+\beta_{j}+\varphi_{k}+s_{p\left(j\right)}+e_{ijkp}$$

where, $y_{ijkp}$ observed [ex: (AUEC_PL 5–240_ and E_max PL_)] µ overall mean

$\alpha_{i}$ effect of the i^th^ investigational product (IP), a fixed effect $\beta_{j}$ effect of the j^th^ sequence, a fixed effect $\varphi_{k}$ effect of the k^th^ period, fixed effect $s_{p\left(j\right)}$ effect of the p^th^ participant nested within the j^th^ sequence, assumed i. i.d. $N\left(0,\sigma_{S}^{2}\right)$ , a random effect $e_{ijkp}$ random error, assumed i. i.d. $N\left(0,\sigma^{2}\right)$

Positive PE (AUEC_PEpos 5–240_ and E_max PEpos_), negative PE (AUEC_PEneg 5–240_ and E_max PEneg_), OPL (E_overall PL_), and OIUA (E_overall IUA_) endpoints for each Velo ONP were compared to those for both the high- and low-AL comparators by an ANOVA as specified in the model above. Scores for UTS measures (AUEC_UTS 0–15_ and AUEC_UTS 0–240_), in addition to the minimum effect of UTS (E_min UTS_), for each Velo ONP were compared to those for the high- and low-AL comparators using a mixed-effects analysis of covariance (ANCOVA). For all secondary endpoints, an alpha level of ≤0.05 was considered significant. The mixed effects ANCOVA model was specified as follows:

$$y_{ijkp}=µ+\alpha_{i}+\beta_{j}+\varphi_{k}+s_{p\left(j\right)}+e_{ijkp}$$

where,

$y_{ijkp}$ observed [ex: (AUEC or E_max_)]

µ overall mean

$\alpha_{i}$ effect of the i^th^ investigational product (IP), a fixed effect

$\beta_{j}$ effect of the j^th^ sequence, a fixed effect

$\varphi_{k}$ effect of the k^th^ period, fixed effect

ξ common slope of baseline UTS, a continuous covariate; $x_{ip}$ baseline value of UTS of the i^th^ IP and the p^th^ subject, a fixed effect;

$s_{p\left(j\right)}$ effect of the p^th^ participant nested within the j^th^ sequence, assumed i. i.d. $N\left(0,\sigma_{S}^{2}\right)$ , a random effect

$e_{ijkp}$ random error, assumed i. i.d. $N\left(0,\sigma^{2}\right)$

A mixed-effects model ANOVA was used to compare nicotine PK parameters (AUC_0-15_, AUC_0-240_, and C_max_) for each Velo ONP to those for the high- and low-AL comparators, while a Wilcoxon nonparametric signed rank test was used for comparisons of T_max_ for each Velo ONP and the high- and low-AL comparators. Data for AUC_0-15_, AUC_0-240_, and C_max_ were analyzed on the natural log scale; T_max_ was analyzed on the original scale. An alpha level of 0.05 was used for all PK parameters assessed. No inferential statistical comparisons were performed between any of the Velo ONPs. The mixed effects model for the analysis of PK parameters (AUC and C_max_) was specified as follows:

$$y_{ijkp}=µ+\alpha_{i}+\beta_{j}+\varphi_{k}+s_{p\left(j\right)}+e_{ijkp}$$

where, $y_{ijkp}$ observed [e.g., ln (AUC or C_max_)] µ overall mean

$\alpha_{i}$ effect of the i^th^ investigational product, a fixed effect $\beta_{j}$ effect of the j^th^ sequence, a fixed effect $\varphi_{k}$ effect of the k^th^ period, fixed effect $s_{p\left(j\right)}$ effect of the p^th^ subject nested within the j^th^ sequence, assumed i. i.d. $N\left(0,\sigma_{S}^{2}\right)$ , a random effect $e_{ijkp}$ random error, assumed i. i.d $N\left(0,\sigma^{2}\right)$

An example of the code used for statistical analysis of T_max_ is given below:

PROC NPAR1WAY WILCOXON;
    CLASS PRODUCT;
    VAR T_max_;
Run;

## Results

### Study populations

In total, 41 participants were enrolled in Study 1, 43 in Study 2, and 36 in Study 3. Of these, 40 completed Studies 1 and 2 each, and 35 completed Study 3 (Supplementary Figure S1). One participant in Study 1 was withdrawn due to an AE. Three participants in Study 2 were withdrawn: one due to an AE, one due to failing to meet continuation criteria, and one due to undisclosed reasons. One participant in Study 3 was withdrawn due to an AE.

Overall, the study populations were predominantly white (Study 1, 78.0%; Study 2, 65.1%; and Study 3, 83.3%) and male (63.4%, 76.7%, and 69.4%, respectively) (Table 2). Across the three studies, participants were long-term cigarette smokers who had smoked on average 15.8–18.0 cigarettes per day for an average range of 20.9–23.9 years. Those who also used STPs accounted for 22.0%, 44.2%, and 36.1% of participants in Studies 1, 2 and 3, respectively. The overall level of cigarette dependence at baseline was moderate, based on FTND scores (mean total scores ranged from 5.9 to 6.3).

**TABLE 2:** Summary of participant demographics and characteristics (safety population)*.

Characteristic	Study		
	Study 1 Velo Pouch AL N = 41	Study 2 Velo Mini Pouch AL N = 43	Study 3 Velo Pouch PK N = 36
Age (years)	42.4 ± 10.3	40.6 ± 8.1	40.4 ± 9.1
Weight (kg)	82.8 ± 21.1	94.3 ± 22.7	90.9 ± 20.3
Height (cm)	171 ± 8.1	174 ± 10.6	173 ± 8.4
BMI (kg/m^2^)	28.3 ± 6.6	31.0 ± 6.7	30.3 ± 6.0
Gender			
Male	26 (63.4)	33 (76.7)	25 (69.4)
Female	15 (36.6)	10 (23.3)	11 (30.6)
Ethnicity			
Hispanic/Latino	14 (34.1)	0	1 (2.8)
Not Hispanic/Latino	27 (65.9)	43 (100)	35 (97.2)
Not reported			
Race			
White	32 (78.0)	28 (65.1)	30 (83.3)
Black/African American	8 (19.5)	14 (32.6)	5 (13.9)
Multiple/Other	1 (2.4)	1 (2.3)	1 (2.8)
Smoking status			
Number of years smoked	20.9 ± 12.5	23.9 ± 8.4	23.8 ± 9.9
Cigarettes smoked per day	15.8 ± 5.5	18.0 ± 6.9	16.6 ± 5.9
FTND score	6.3 ± 1.6	5.9 ± 1.4	5.9 ± 1.5
Smokers who also used ST	9 (22.0)	19 (44.2)	13 (36.1)

*Values are given as mean ± SD, or number (percentage).

Abbreviations: AL, abuse liability; BMI, body mass index; FTND, Fagerström Test for Nicotine Dependence; n, number of observations; N, number of participants; PK, pharmacokinetic; SD, standard deviation; ST, smokeless tobacco.

### Nicotine pharmacokinetics and subjective effects of Velo oral nicotine pouches (Studies 1 and 2)

In Studies 1 and 2, we evaluated the AL of Velo Pouches and Velo Mini Pouches, respectively, differing in nicotine level and flavor by measuring nicotine pharmacokinetics and subjective effects during product use.

#### Nicotine pharmacokinetics

**FIGURE 2:**
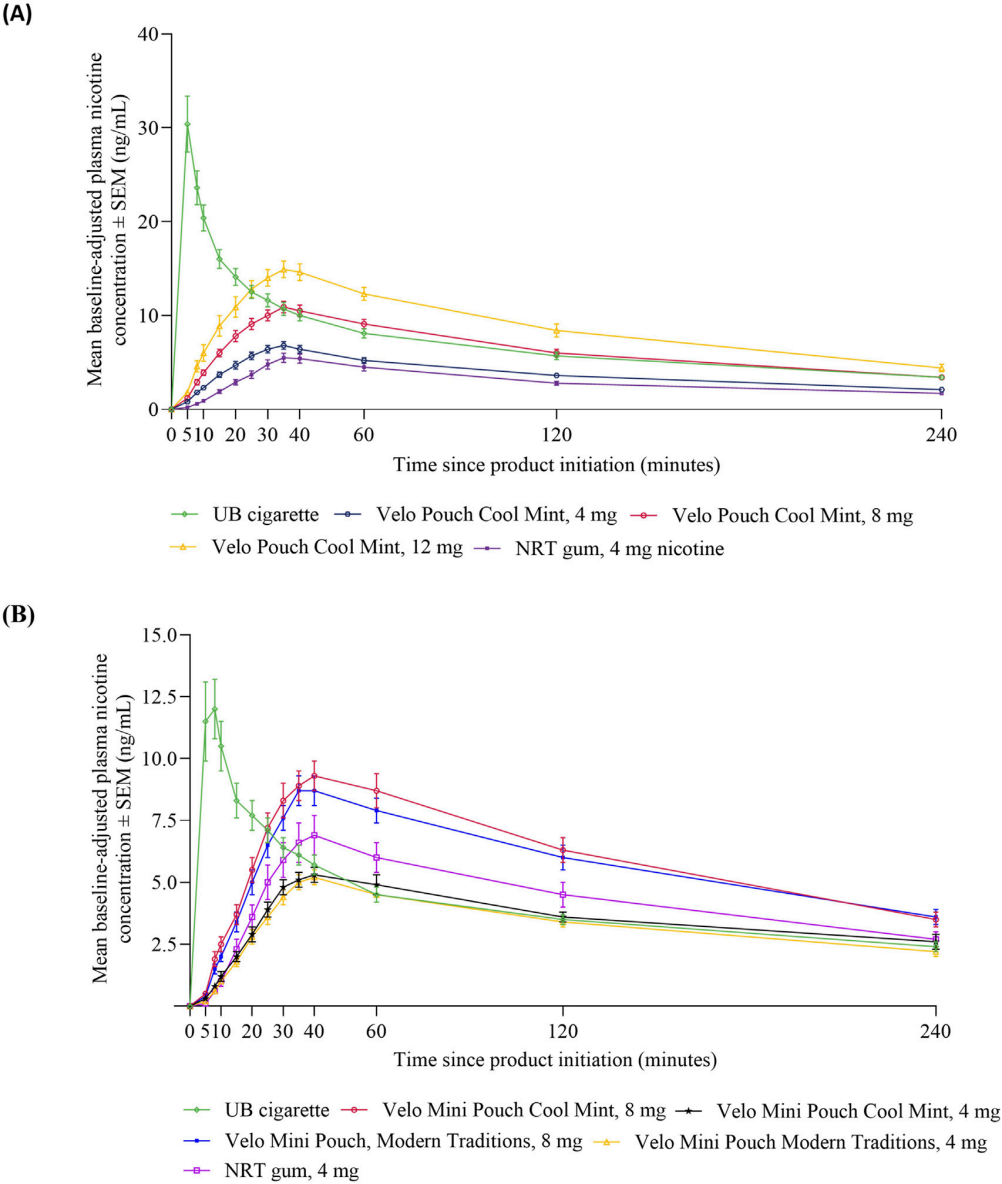
Plasma nicotine concentrations over time for Velo ONPs evaluated in Studies 1 and 2. Each point shows the mean ± SEM plasma nicotine concentration in Study 1 **(A)** and Study 2 **(B)**. Abbreviations: AL, abuse liability; mg, milligram (of nicotine); ng/mL, nanograms per milliliter; NRT, nicotine replacement therapy; SEM, standard error of the mean; UB, usual brand; Velo ONP, Velo oral nicotine pouches.

Mean baseline-adjusted plasma nicotine concentrations over time following use of the study products are presented in Figure 2. The nicotine PK profiles were similar across the Velo ONPs in both Study 1 (Figure 2A) and Study 2 (Figure 2B), with plasma nicotine levels increasing shortly after the start of product use, peaking at ∼35 min, and decreasing gradually thereafter. By contrast, use of UB cigarette led to a sharp rise in plasma nicotine, which peaked at ∼5 min and then declined more rapidly. The PK profile of the NRT gum was similar to the Velo ONPs.

The statistical comparisons and test statistics for the PK parameters of Velo ONPs and comparator products are presented in Table 3 and Supplementary Table S1, respectively. For the Velo Pouches in Study 1, nicotine uptake parameters (AUC_0-15_, AUC_0-240_, and C_max_) increased with increasing product nicotine content, were significantly lower than those of UB cigarette, and were each significantly higher than those of NRT gum (Table 3). The only exception was the Velo Pouch with the highest nicotine content (12 mg), which had an overall nicotine uptake (AUC_0-240_) that was not significantly different from UB cigarette. Median T_max_ was significantly longer for Velo Pouches than for UB cigarette but did not differ between any of the Velo Pouches and NRT gum (Table 3; Supplementary Table S1), consistent with the use periods of these product types.

**TABLE 3:** Results of nicotine uptake parameters of Velo ONPs* evaluated in Studies 1 and 2.

Parameter	Statistic	Study 1: Velo Pouch AL					Study 2: Velo Mini Pouch AL					
		Cool Mint (4 mg)	Cool Mint (8 mg)	Cool Mint (12 mg)	UB cig	NRT gum	Cool Mint (4 mg)	Cool Mint (8 mg)	Modern Traditions (4 mg)	Modern Traditions (8 mg)	UB cig	NRT gum
		N = 40	N = 41	N = 40	N = 40	N = 40	N = 42	N = 42	N = 42	N = 42	N = 42	N = 42
AUC_0-15_ (ng x min/mL)	Mean	20.3^a^ ^,^ ^b^	35.1^a^ ^,^ ^b^	46.8^a^ ^,^ ^b^	268.6	8.2	9.2^a^	20.2^a^ ^,^ ^b^	8.4^a^	16.8^a^ ^,^ ^b^	97.8	7.4
	95% CI Lower	16.5	28.3	37.6	215.9	6.6	6.9	15.1	6.3	12.6	73.4	5.6
	95% CI Upper	25.1	43.6	58.3	334.1	10.1	12.2	27.0	11.2	22.4	130.3	9.8
AUC_0-240_ (ng x min/mL)	Mean	805.6^a^ ^,^ ^b^	1305.7^a^ ^,^ ^b^	1763.1^b^	1680.5	648.2	791.8	1304.0^a^ ^,^ ^b^	714.3^a^	1221.5^a^ ^,^ ^b^	881.1	804.4
	95% CI Lower	701.7	1134.8	1528.6	1457.8	563.9	677.6	1112.0	611.2	1041.8	753.0	689.3
	95% CI Upper	924.8	1502.4	2033.6	1937.1	745.2	925.3	1529.1	834.8	1432.2	1031.1	938.6
C_max_ (ng/mL)	Mean	6.6^a^ ^,^ ^b^	10.5^a^ ^,^ ^b^	14.8^a^ ^,^ ^b^	27.5	5.2	5.9^a^	9.4^b^	5.2^a^	9.0^b^	10.8	5.9
	95% CI Lower	5.7	9.1	12.7	23.6	4.5	5.0	7.9	4.4	7.5	9.1	5.0
	95% CI Upper	7.6	12.2	17.2	32.0	6.1	7.1	11.2	6.2	10.7	12.9	7.1
T_max_ (min)	Median	35.0^a^	35.0^a^	35.1^a^	5.1	35.0	40.0^a^	40.0^a^	39.0^a^	39.0^a^	7.0	39.5
	Minimum	30.0	35.0	34.9	5.0	34.8	35.0	35.0	35.0	35.0	5.0	35.0
	Maximum	40.0	40.0	40.0	8.0	40.0	60.0	60.0	59.0	59.0	7.0	60.0

*Values are reported as least-squares mean (AUC and C_max_) and median (T_max_).

^a^ Significantly different (p < 0.05) vs UB cigarette.

^b^ Significantly different (p < 0.05) vs. NRT, gum.

Abbreviations: AL, abuse liability; AUC, area under the curve; AUC_0-15_, AUC, for 0–15 min after initiation of product use; AUC_0-240_, AUC, for 0–240 min after initiation of product use; CI, confidence interval; C_max_, maximum baseline-adjusted plasma nicotine concentration; min, minutes; mL, milliliter; N, number of participants; ng, nanogram; NRT, nicotine replacement therapy; ONPs, oral nicotine pouches; T_max_, time to reach C_max_, UB, usual brand.

In line with results from Study 1, nicotine uptake parameters (AUC_0-15_, AUC_0-240_, and C_max_) were higher in Study 2 for the two Velo Mini Pouches with higher nicotine content (8 mg) than Velo Mini Pouch products with lower nicotine content (4 mg) (Table 3). Early nicotine uptake, within 15 min of product use (AUC_0-15_), was significantly lower for each Velo Mini Pouch than for UB cigarette. Nicotine uptake over 4 h (AUC_0-240_) was significantly higher for both 8 mg Velo Mini Pouch (Cool Mint and Modern Traditions) products, but significantly lower for the 4 mg Velo Mini Pouch (Modern Traditions) product, when compared to UB cigarette. No significant difference was observed in AUC_0-240_ between the Velo Mini Pouch Cool Mint 4, mg and UB cigarette. The C_max_ values for both Velo Mini Pouches in 8 mg were similar regardless of flavor and did not differ significantly from UB cigarette. However, C_max_ for the Velo Mini Pouches in 4 mg was significantly lower than that for UB cigarette (Table 3; Supplementary Table S1).

Relative to NRT gum, AUC_0-15_, AUC_0-240_, and C_max_ were not different for the Velo Mini Pouch 4 mg products but were significantly higher for the Velo Mini Pouch 8 mg products. As in Study 1, median T_max_ was significantly longer for all Velo Mini Pouches than for UB cigarette but did not differ between any of the Velo Mini Pouches and NRT gum (Table 3; Supplementary Table S1).

#### Subjective effects

The participants in the two AL studies self-assessed their experience of using the Velo ONPs by five subjective effects questionnaires. In general, UB cigarettes were rated most favorably among the study products, as described below. Data on statistical comparisons of subjective effects parameters are summarized in Tables 4, 5, with t-statistics and p-values provided in Supplementary Table S2.

**TABLE 4:** Summary of product liking and urge to smoke subjective effects measures of Velo ONPs^a^ evaluated in Studies 1 and 2.

Parameter	Statistic	Study 1: Velo Pouch AL					Study 2: Velo Mini Pouch AL						
		Cool Mint (4 mg)	Cool Mint (8 mg)	Cool Mint (12 mg)	UB cig	NRT gum	Cool Mint (4 mg)	Cool Mint (8 mg)	Modern Traditions (4 mg)		Modern Traditions (8 mg)	UB cig	NRT gum
		N = 40	N = 41	N = 40	N = 40	N = 40	N = 42	N = 42	N = 42		N = 42	N = 42	N = 42
AUEC_PL 5-240_	Mean	1297.0^a^	1259.7^a^	1250.9^a^	1919.7	1306.9	1026.5^a^	1031.9^a^	1049.4^a^		1071.6^a^	1885.2	118.0
	95% CI Lower	1116.7	1081.3	1070.6	1739.4	1126.5	819.6	826.3	843.8		866.0	1679.8	980.3
	95% CI Upper	1477.4	1438.1	1431.2	2099.9	1487.3	1233.4	1237.4	1255.0		1277.2	2090.6	1388.6
E_max PL_	Mean	6.7^a^	6.7^a^	6.7^a^	9.3	7.1	5.8^a^	5.7^a^	6.4^a^		6.2^a^	9.4	6.5
	95% CI Lower	6.0	6.0	6.0	8.6	6.4	4.9	4.8	5.5		5.3	8.5	5.6
	95% CI Upper	7.4	7.4	7.4	10.0	7.8	6.7	6.6	7.3		7.1	10.3	7.4
AUEC_UTS 0-15_	Mean	91.5^a^	81.7^a^ ^,^ ^b^	84.6^a^	67.2	94.8	104.4^a^	104.2^a^	109.5^a^		108.3^a^	66.7	103.0
	95% CI Lower	82.9	73.2	75.9	58.5	86.2	93.0	92.9	98.2		97.4	55.5	91.8
	95% CI Upper	100.1	90.3	93.3	75.9	103.4	115.7	115.4	120.7		119.6	78.0	114.2
AUEC_UTS 0-240_	Mean	1779.7	1624.0^b^	1616.8^b^	1667.6	1843.3	1818.0^a^	1769.2	1935.2^a^ ^,^ ^b^		1848.1^a^	1692.3	1759.7
	95% CI Lower	1617.4	1462.5	1453.5	1504.5	1681.2	1678.7	1630.6	1796.6		1709.3	1553.8	1621.9
	95% CI Upper	1942.1	1785.6	1780.1	1830.7	2005.4	1957.3	1907.9	2073.8		1986.9	1830.8	1897.6
E_min UTS_	Mean	5.7^a^	4.9^a^ ^,^ ^b^	4.7^b^	3.8	6.1	6.1^a^	5.7^a^	6.5^a^		6.2^a^	3.1	5.9
	Minimum	4.7	4.0	3.7	2.8	5.1	5.2	4.8	5.6		5.3	2.2	5.0
	Maximum	6.7	5.9	5.7	4.8	7.1	7.0	6.6	7.4		7.1	4.0	6.8

*Values are reported as least-squares mean.

^a^ Significantly different (p < 0.0042 [Study 1]; p < 0.0031 [Study 2] for PL parameters, and p < 0.05 for UTS parameters) vs UB cigarette.

^b^ Significantly different (p < 0.0042 [Study 1]; p < 0.0031 [Study 2] for PL, parameters, and p < 0.05 for UTS, parameters) vs. NRT, gum.

Abbreviations: AL, abuse liability; AUEC, area-under-the-effect curve; AUEC_PL5-240,_ AUEC, for PL, for 5–240 min after the start of product use; AUEC_UTS, 0–240,_ AUEC, for UTS, for 0–240 min following initiation of product use; AUEC_UTS, 0–15,_ AUEC, for UTS, for 0–15 min following initiation of product use; CI, confidence interval; E_max PL_, the maximum PL, effect after the start of product use; E_min UTS_, minimum UTS; N, number of participants; NRT, nicotine replacement therapy; PL, product liking; UB, usual brand; UTS, urge to smoke.

**TABLE 5:** Summary of subjective measures of product effects, overall product liking, and overall intent to use again of Velo ONPs* evaluated in Studies 1 and 2.

Parameter	Statistic	Study 1: Velo Pouch AL					Study 2: Velo Mini Pouch AL					
		Cool Mint (4 mg)	Cool Mint (8 mg)	Cool Mint (12 mg)	UB cig.	NRT gum	Cool Mint (4 mg)	Cool Mint (8 mg)	Modern Traditions (4 mg)	Modern Traditions (8 mg)	UB cig	NRT gum
		N = 40	N = 41	N = 40	N = 40	N = 40	N = 42	N = 42	N = 42	N = 42	N = 42	N = 42
AUEC_PEpos 5-240_	Mean	1048.0^a^	960.1^a^	1009.9^a^	1420.0	865.7	852.8^a^	836.9^a^	778.5^a^ ^,^ ^b^	857.2^a^	1403.0	1002.6
	95% CI Lower	818.3	732.6	780.4	1190.5	636.0	604.2	589.4	531.0	609.7	1155.7	756.4
	95% CI Upper	1277.6	1187.6	1239.4	1649.5	1095.3	1101.4	1084.4	1026.0	1104.7	1650.4	1248.8
E_max PEpos_	Mean	6.5^a^	6.5^a^	6.6^a^	9.0	6.2	5.6^a^	5.3^a^	5.3^a^	5.4^a^	8.9	5.9
	95% CI Lower	5.5	5.6	5.6	8.0	5.2	4.8	4.3	4.3	4.4	7.9	4.9
	95% CI Upper	7.4	7.5	7.6	9.9	7.2	6.8	6.3	6.3	6.4	9.9	6.9
AUEC_PEneg 5–240_	Mean	334.0	387.0	398.2	334.7	259.8	401.4	517.1^b^	449.7	372.6	301.0	424.2
	95% CI Lower	174.7	229.0	238.9	175.5	100.5	214.9	331.6	264.2	187.0	115.6	239.8
	95% CI Upper	493.3	544.9	557.4	494.0	419.1	588.0	702.6	635.2	558.0	486.4	608.6
E_max PEneg_	Mean	2.9	3.2	3.9^b^	3.5	2.2	2.9	3.3	3.2	3.2	2.6	2.6
	95% CI Lower	1.9	2.2	2.9	2.4	1.2	1.8	2.2	2.1	2.1	2.1	1.5
	95% CI Upper	3.9	4.2	4.9	4.5	3.2	4.0	4.4	4.3	4.3	4.3	3.7
E_overall PL_	Mean	6.5^a^	6.0^a^	5.3^a^	8.6	5.7	4.9^a^	4.8^a^	4.6^a^ ^,^ ^b^	4.3^a^ ^,^ ^b^	9.0	5.6
	Minimum	5.7	5.7	4.5	7.8	4.8	4.0	3.8	3.6	2.1	8.1	4.6
	Maximum	7.4	7.4	6.2	9.4	6.5	5.9	5.7	5.5	4.3	9.9	6.5
E_overall IUA_	Mean	5.0^a^	4.7^a^	4.2^a^	9.0	4.6	4.0^a^ ^,^ ^b^	4.0^a^	3.5^a^ ^,^ ^b^	3.7^a^ ^,^ ^b^	9.1	5.0
	Minimum	3.9	3.6	3.2	7.9	3.5	2.9	3.0	2.5	2.7	8.1	4.0
	Maximum	6.1	5.8	5.3	10.0	5.7	5.0	5.1	4.5	4.7	10.1	6.0

*Data are reported as least-squares mean.

^a^ Significantly different (p < 0.05) vs UB cigarette.

^b^ Significantly different (p < 0.05) vs. NRT, gum.

Abbreviations: AUEC_PEpos, 5–240,_ AUEC, for positive PE, for 5–240 min after the start of product use; AL, abuse liability; AUEC_PEneg, 5–240,_ AUEC, for negative PE, for 5–240 min after the start of product use; CI, confidence interval; E_max PEneg,_ maximum positive PE; E_max PEpos_, maximum positive PE; E_overall IUA_, effect of overall intent to use again at 240 min after product use; E_overall PL_-effect of overall PL, at 240 min after product use; N, number of participants; NRT, nicotine replacement therapy; PE, product effects; PL, product liking; UB, usual brand.

##### Product liking

Among all study products, UB cigarettes were rated highest for the two PL parameters, AUEC_PL 5–240_ and E_max PL_ (Table 4). Values for these parameters were significantly lower for all Velo ONPs than for UB cigarette in both AL studies but did not differ between the Velo ONPs and NRT gum (Table 4; Supplementary Table S2).

##### Urge to smoke

**FIGURE 3:**
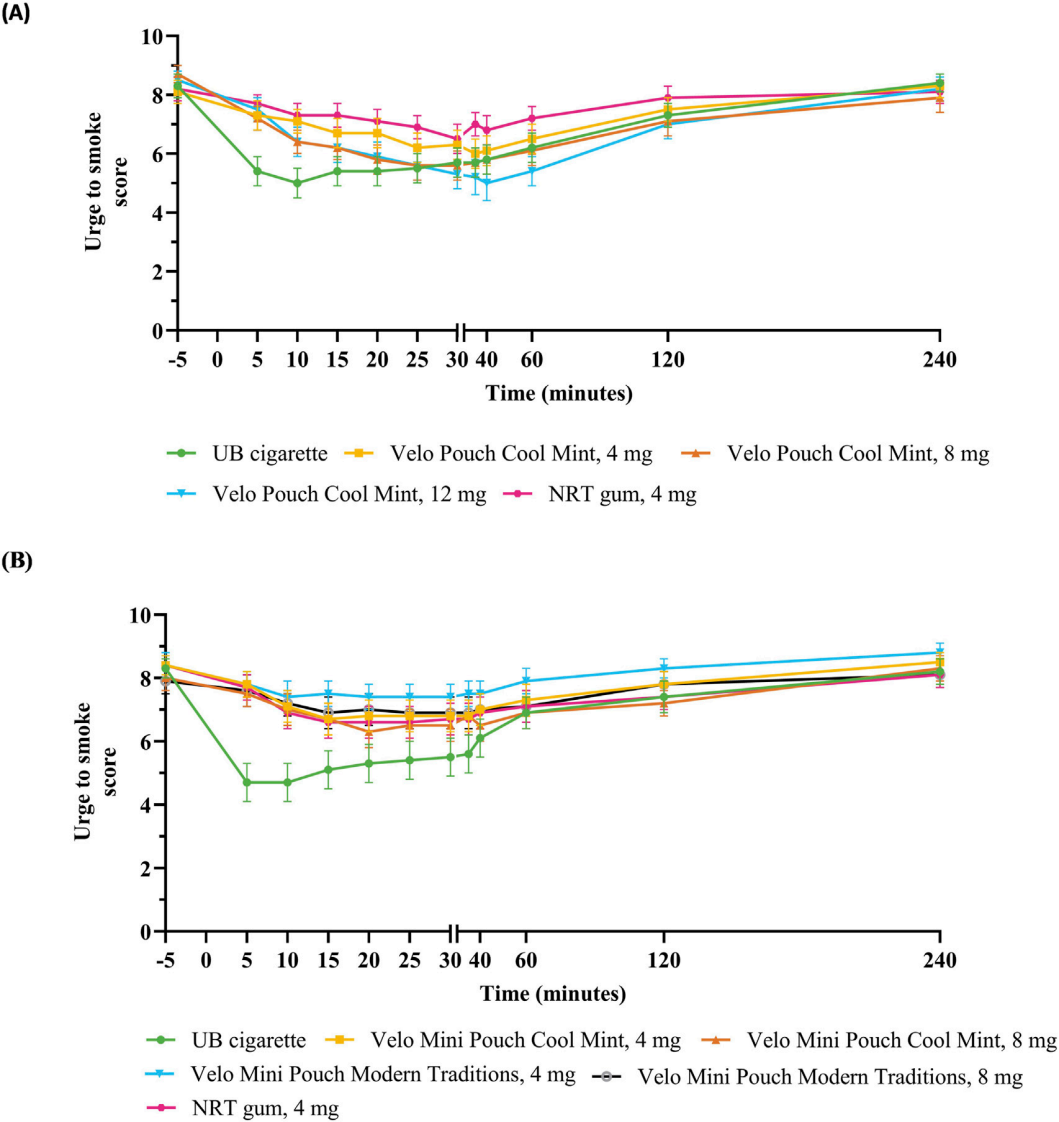
Urge to smoke scores over 240 min after initiation of product use. Each point shows the mean ± SEM in Study 1 **(A)** and Study 2 **(B)**. Abbreviations: AL, abuse liability; mg, milligram (of nicotine); NRT, nicotine replacement therapy; SEM, standard error of the mean; UB, usual brand.

Evaluation of UTS in both AL studies indicated that UB cigarette reduced smoking urges within the first 15 min of product use (AUEC_UTS 0–15_) to a significantly greater extent than the Velo ONPs irrespective of nicotine content, pouch size or flavor (Table 4; Figures 3A, B). In Study 1, AUEC_UTS 0–240_ and E_min UTS_ for the Velo Pouches with the highest nicotine content (12 mg), were not significantly different to those for UB cigarette, while the Velo Pouches in 4 mg reduced UTS (AUEC_UTS 0–15_, AUEC_UTS 0–240_, and E_min UTS_) to a similar extent as NRT gum (Table 4; Supplementary Table S2). In general, the higher the level of nicotine in the Velo Pouch, the lower the UTS score compared with NRT gum.

In Study 2, the overall UTS (AUEC_UTS 0–240_) for each Velo Mini Pouch was significantly greater compared with UB cigarettes; the exception was AUEC_UTS 0–240_ for the Velo Mini Pouch Cool Mint 8 mg product, which did not differ significantly from UB cigarette. In addition, the minimum effect of each Velo Mini Pouch to relieve UTS (E_min UTS_) was significantly greater for each Velo Mini Pouch than for UB cigarette. Relative to NRT gum, each Velo Mini Pouch relieved UTS (AUEC_UTS 0–15_, AUEC_UTS 0–240_, and E_min UTS_) to a similar extent as NRT gum, except that AUEC_UTS 0–240_ for the Velo Mini Pouch Modern Traditions, 4 mg was significantly greater than that for NRT gum. Both AUEC_UTS 0–240_ and E_min UTS_ were lower for the 8 mg Velo Mini Pouch than for the Velo Mini Pouch, 4 mg (Table 4; Supplementary Table S2).

##### Product effects

Parameters of both positive (AUEC_PEpos 5–240_ and E_max PEpos_) and negative (AUEC_PEneg 5–240_ and E_max PEneg_) PE were assessed. Regarding positive PE, UB cigarette was rated highest in both Studies 1 and 2, and scores were significantly higher for UB cigarette than for any Velo ONP. The NRT gum and the Velo ONPs evaluated received comparable scores for positive PE parameters in both studies; the exception was AUEC_PEpos 5–240_, which was significantly lower for the Velo Mini Pouch Modern Traditions, 4 mg than for NRT gum (Table 5; Supplementary Table S2).

Both overall (AUEC_PEneg 5–240_) and maximum (E_max PEneg_) negative PE did not differ significantly between any of the Velo ONPs and UB cigarette or NRT gum with two exceptions (Table 5). First, the highest nicotine Velo Pouch (12 mg) product was scored significantly higher than NRT gum for E_max PEneg_; and second, the Velo Mini Pouch Cool Mint, 8 mg product was scored significantly higher than UB cigarettes for AUEC_PEneg 5–240_. In general, among the Velo ONPs, negative PE increased with higher nicotine content (Table 5; Supplementary Table S2).

##### Overall product liking

In both AL studies, E_overall PL_ was significantly lower for all Velo ONPs relative to UB cigarette (Table 5), whereas E_overall PL_ for NRT gum and Velo ONPs was generally comparable with the exception that significantly lower E_overall PL_ values were observed between the Velo Mini Pouches Modern Traditions (4 and 8 mg) and NRT gum (Table 5; Supplementary Table S2).

##### Overall intent to use again

Consistent with the other subjective effects measures, OIUA was highest for UB cigarette among the study products (Table 5). E_overall IUA_ values for all Velo ONPs were significantly lower than for UB cigarette in both AL studies and did not differ significantly between the Velo ONPs and NRT gum in Study 1. Although E_overall IUA_ tended to be higher irrespective of flavor for the 8 mg *versus* the 4 mg Velo Mini Pouch products in Study 2, E_overall IUA_ was significantly lower for the Velo Mini Pouch products than for NRT gum, except for the Velo Mini Pouch Cool Mint, 8 mg, where it was observed that E_overall IUA_ did not differ from that of NRT gum (Table 5; Supplementary Table S2).

In summary, key subjective effects measures (i.e., PL, OPL, positive PE, and OIUA) for the Velo ONPs in both AL studies were consistently significantly lower compared with UB cigarette, and similar to or slightly lower than the respective measures for NRT gum.

### Nicotine pharmacokinetics of Velo oral nicotine pouches (Study 3)

**FIGURE 4:**
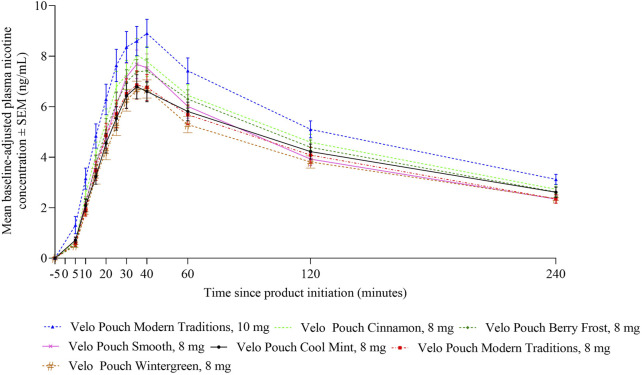
Plasma nicotine concentrations over 240 min following start of Velo Pouch product use in Study 3. Each point shows the mean ± SEM plasma nicotine concentration. Abbreviations: mg, milligram (of nicotine); PK, pharmacokinetic; SEM, standard error of the mean.

In Study 3, we evaluated whether nicotine uptake and OPL differ between different Velo Pouch flavors at the same nicotine level. As in the two AL studies, nicotine PK profiles were generally similar for all Velo Pouches at the same nicotine level. The plasma nicotine level for the 10 mg product was higher than for the 8 mg products (Figure 4). Overall plasma nicotine uptake (AUC_0-240_) and maximum plasma nicotine concentration (C_max_) were generally similar for all flavors of the 8 mg Velo Pouch nicotine products, and higher for the 10 mg nicotine product (Table 6). Additionally, the pharmacokinetic parameters (AUC_0-15_, AUC_0-240_, and C_max_) for the 8 mg products generally exhibited overlapping 95% confidence intervals (Table 6).

**TABLE 6:** Results of nicotine uptake and overall product liking scores of Velo Pouches* assessed in Study 3.

Parameter	Statistic	Velo Pouch						
		Modern Traditions (10 mg)	Modern Traditions (8 mg)	Cool Mint (8 mg)	Berry Frost (8 mg)	Cinnamon (8 mg)	Wintergreen (8 mg)	Smooth (8 mg)
		N = 35	N = 35	N = 36	N = 35	N = 36	N = 36	N = 36
AUC_0-15_ (ng × min/mL)	n	33	35	35	34	36	36	36
	Mean	34.4	21.1	21.3	22.6	25.8	19.6	23.8
	95% CI Lower	19.9	13.1	14.3	15.0	13.4	14.1	14.7
	95% CI Upper	34.4	21.3	22.2	23.5	25.0	20.4	24.3
AUC_0-240_ (ng × min/mL)	n	33	35	35	34	36	36	36
	Mean	1259.4	955.7	991.2	1054.6	1097.4	907.2	988.1
	95% CI Lower	1036.7	741.4	799.3	883.0	847.1	721.6	760.4
	95% CI Upper	1349.1	1018.6	1059.4	1125.1	1178.2	972.9	1059.8
C_max_ (ng/mL)	n	33	35	35	34	36	36	36
	Mean	9.8	7.5	7.3	8.0	8.7	7.1	8.2
	95% CI Lower	7.9	5.8	6.1	6.7	6.7	5.8	6.4
	95% CI Upper	10.4	8.0	7.8	8.5	9.2	7.6	8.7
T_max_ (min)	n	33	35	35	34	36	36	36
	Median	35	35	35	35	35	35	35
	Minimum	20	20	25	20	25	25	25
	Maximum	60	60	120	60	120	60	60
^b^E_overall PL_	Mean	5.1	5.4	5.7	5.9	5.3	5.5	5.8
	SD	2.3	2.4	2.2	2.1	2.6	2.2	1.8
	Minimum	0.0	1.0	0.0	0.0	0.0	0.0	2.0
	Maximum	10.0	10.0	9.0	10.0	10.0	8.0	10.0

*Means are reported for AUC, C_max_, and E_overall PL_, and medians are reported for T_max_.

Abbreviations: AUC, area under the curve; AUC_0-15,_ AUC, for 0–15 min after initiation of product use; AUC_0-240,_ AUC, for 0–240 min after initiation of product use; C_max_, maximum baseline-adjusted plasma nicotine concentration; CI, confidence interval; E_overall PL_, effect of overall PL; mg, milligram (of nicotine); N, number of participants for each parameter; n, number of observations; PL, product liking; SD, standard deviation; T_max_, time to reach C_max_.

Overall product liking was rated at the end of each test session and was similar for all Velo Pouches regardless of flavor or nicotine content (Table 6).

### Adverse events in the three study populations

In all three studies, all AEs, their causal relationship (related or possibly related) to use of the study products, and their severity (mild, moderate, or severe) were recorded (Supplementary Tables S3A–C). Few participants experienced an AE; in general, AEs were transient, and the majority were mild. No participants experienced a severe AE (Supplementary Tables S3A–C).

Nausea, dizziness, hiccups, headache, and euphoric mood were the most reported AEs during use of the Velo ONPs (Supplementary Table S4). One participant in Study 1 withdrew early due to two AEs (vascular disorders: diastolic and systolic hypertension), both mild in severity. Diastolic hypertension was judged unlikely to be related to use of the Velo Pouch, while systolic hypertension was reported as possibly related to use of the Velo Pouch Cool Mint, 8 mg product. One participant in Study 1 was discontinued early due to five AEs; one AE (hypertension) was moderate in severity, while the others (hyperesthesia, tachycardia, and flushing [one case of each], and nausea [two cases]) were mild. One case of nausea, hypertension, tachycardia, and flushing were judged as possibly related to use of NRT gum. The other case of nausea was judged as related to the use of the Velo Mini Pouch Modern Traditions, 4 mg, while the case of hyperesthesia was judged as possibly related to use of Velo Mini Pouch Cool Mint, 8 mg. All AEs deemed to be causally related to product use were resolved prior to participant discharge from the study.

## Discussion

The objective of the three clinical studies described here was to provide a comprehensive and rigorous assessment of the elements contributing to the AL of Velo ONPs through measurements of nicotine PK (exposure) and subjective effects (Carter et al., 2009b; Fearon et al., 2022; Henningfield and Keenan, 1993; Vansickel et al., 2022). The key findings are as follows: (1) nicotine uptake increased with increasing nicotine content in the Velo ONPs, and PK parameters across different flavors at the same nicotine level were largely similar; (2) subjective effects for Velo ONPs were generally lower relative to UB cigarette and were generally similar across all Velo ONPs variants, showing comparable or lower subjective effects to NRT gum; (3) Velo ONPs reduced UTS, with greater reductions observed for higher nicotine content pouches, though still less effective than cigarettes; and (4) Velo ONPs were well tolerated by the participants in the studies.

Collectively, our findings suggest that Velo ONPs deliver sufficient nicotine to users to maintain reinforcement by reducing smoking urges, and exhibit some AL, but to a lesser extent than combustible cigarettes. Since a certain degree of dependence is necessary for alternative nicotine products to effectively provide a viable substitute for cigarettes (Abrams et al., 2018; Fearon et al., 2022), these findings suggest that Velo ONPs could be a viable component of a THR strategy. Notably, the slower nicotine uptake, reflected by the later T_max_, differences in the AUC_0-15_, and the overall lower AL of Velo ONPs observed here, suggest that these products pose less of an initiation and/or addiction risk among non-tobacco users. Additionally, the reduced levels of HPHCs (Grandolfo et al., 2024; Back et al., 2023; Borgerding et al., 2012; Azzopardi et al., 2022a; Jablonski et al., 2022) and reduced biomarkers of exposure (Grandolfo et al., 2024; Azzopardi et al., 2022b) in ONPs further support that Velo ONPs may play a contributory role in THR and a benefit to the population as a whole, building on the previously demonstrated positive impact on public health of oral STPs (Clarke et al., 2019; Food and Drug Administration, 2019; Food and Drug Administration, 2023).

Consistent with the delivery of nicotine via buccal absorption, the PK profiles of Velo ONPs resembled those of NRT gum, with a significantly higher T_max_ and lower C_max_ compared with UB cigarette. In Study 1, the C_max_ and overall nicotine uptake after Velo Pouch use was associated with nicotine content level, in agreement with other studies in which ONPs with a high nicotine content exhibited C_max_ and AUC comparable to or higher than those of cigarettes (Keller-Hamilton et al., 2023; Liu et al., 2021; McEwan et al., 2022a) and traditional STPs (Lunell et al., 2020). The Velo Mini Pouches (4 and 8 mg) also exhibited nicotine content-dependent C_max_ and AUC values. The highest nicotine level (12 mg) product evaluated in Study 1, although comparable to UB cigarette in terms of overall nicotine uptake, did not achieve a comparable C_max_ value to that of UB cigarette. In addition, the Velo Pouch Cool Mint 4 mg product exhibited significantly higher C_max_ and AUC values as compared with NRT gum in Study 1, whereas in Study 2, the same nicotine content product (Velo Mini Pouch Cool Mint 4 mg) was not different than NRT gum; but the 8 mg product had significantly higher C_max_ and AUC values than NRT gum. These findings suggest that other characteristics, such as formulation and composition (Peraza et al., 2024), user behavior (Digard et al., 2012), and individual differences (Wagenknecht et al., 2018) of ONPs, and not just the nicotine content, may influence nicotine PK.

The plasma nicotine AUC_0-240_ and C_max_ values for the 8 mg products of the same flavor (Cool Mint) were similar across Studies 1 and 2, indicating consistent nicotine delivery from Velo ONPs. In contrast, the AUC_0-240_ and the C_max_ values for UB cigarette were higher in Study 1 than in Study 2. In Study 2, AUC_0-240_ was significantly higher for both 8 mg Velo Mini Pouch products (Cool Mint and Modern Traditions) compared to UB cigarette. However, in Study 1, the AUC_0-240_ for Velo Pouch Cool Mint at the same nicotine level (8 mg) was significantly lower compared to UB cigarette. Variability in nicotine uptake from cigarettes has been previously reported (Hammond et al., 2005; Krebs et al., 2016), and may account for the differences in statistical significance when comparing the 8 mg Velo ONPs to UB cigarettes in these two studies. While Velo ONPs are demonstrated to effectively deliver nicotine, albeit at a slower rate compared to cigarettes, the faster nicotine uptake (as indicated by shorter T_max_ and higher AUC _0–15_), the numerically higher peak nicotine levels (C_max_) for the combustible cigarettes assessed in this study compared to the 8 mg Velo Mini Pouch products, support an overall lower AL for the 8 mg Velo Mini Pouch products compared to cigarettes.

We also examined the effect of different flavors on the nicotine PK and AL of Velo ONPs. In Studies 2 and 3, Velo ONPs with different flavors but the same nicotine content generally exhibited similar PK profiles and parameters, indicating that flavor does not affect nicotine PK for the Velo ONPs assessed. In addition, subjective effects scores were generally similar among flavor variants of Velo ONPs; when taken together with the nicotine PK findings, this suggests that flavor alone does not have an impact on AL. Rensch *et al.* (Rensch et al., 2021) previously reported that C_max_ values were within 15%, while AUC values were within 25% of each other for six flavor variants of 4 mg “on!” ONPs. Their results align with our observations that nicotine content is a key determinant of nicotine PK of Velo ONPs, but flavors do not seem to influence nicotine PK, and therefore AL.

In general, the Velo ONPs, irrespective of the pouch size, received lower scores than UB cigarette and comparable to or lower than NRT gum in the subjective measures assessed in the two AL studies. This is consistent with previous studies in which 4 and 8 mg ONPs had lower subjective effects scores than cigarettes (Liu et al., 2022), and where ONPs with various nicotine content all had PL and IUA scores lower than cigarettes (McEwan et al., 2022b). In our AL studies, negative PE scores were generally comparable across the study products. In addition, while cigarettes were most effective in reducing UTS, the study Velo ONPs and NRT gum were also effective, although to a lesser extent than cigarettes, and there was a tendency for the UTS reductions associated with Velo ONP use to be greater with increasing nicotine content. Reductions in UTS have been reported previously for 3 mg and 6 mg ONPs (Keller-Hamilton et al., 2023). When taken together with the current AL findings, this suggests that ONPs, including Velo ONPs, may provide a suitable alternative to cigarettes for current smokers who do not want to quit smoking or using other tobacco and nicotine products.

The main strength of these three studies is the inclusion of several ONPs varying in flavor, nicotine content, and physical pouch size, thus presenting a wide-ranging assessment of the AL of Velo ONPs. In addition, the inclusion of high and low AL comparator products (cigarettes and NRT gum, respectively) enabled relative AL to be determined. A further strength is the use of established methods that conform to proposals and regulatory stipulations on how AL should be assessed (Carter et al., 2009b; Fearon et al., 2022; Henningfield and Keenan, 1993; Vansickel et al., 2022; Campbell et al., 2023; Campbell et al., 2022). It should be noted, however, that the AL determined is representative only of the ONPs assessed and may not extend to other types and brands of ONPs, particularly those with different nicotine contents and flavors. Other limitations include the fact that Study 3 assessed only a single aspect of subjective effects measure. Nevertheless, the findings for OPL in Study 3 agreed with the wider measures evaluated in Studies 1 and 2, indicating a significantly lower potential for ONP adoption compared with cigarettes. Another potential limitation is the length of time for which participants were able to familiarize themselves with the study products (half a day) as it has been shown for other nicotine products (e.g., electronic cigarettes) that user experience may affect nicotine PK (Farsalinos et al., 2015; Hajek et al., 2015) and therefore AL. However, we consider that this is unlikely to be the case for ONPs owing to the simplicity and similarity in product design and instructions for use. Importantly, due to the route of exposure, nicotine uptake for ONPs is expected to be slower than an inhalable product regardless of use behavior.

### Synthesis of evidence for abuse liability determination for Velo ONPs

#### Study 1

This study evaluated elements of AL for Velo Pouch products across three nicotine levels (4 mg, 8 mg, and 12 mg nicotine) in comparison to UB cigarette (a high-AL comparator) and NRT gum (a low-AL comparator) in current smokers and smokers who also use STPs. The findings across subjective and PK endpoints provide a comprehensive basis for determining the relative AL of the Velo Pouch products.

The subjective effects data reveal a clear trend of comparative AL positioned between UB cigarette and NRT gum. Measures of PL showed significantly lower scores for all Velo Pouch products compared to UB cigarette and no significant differences compared to NRT gum. Subjective effects measures, including positive PE, OPL, and OIUA, were consistently lower for Velo Pouch products than for UB cigarette and comparable to NRT gum. Notably, higher nicotine levels were associated with decreased OPL and OIUA scores, suggesting that increased nicotine delivery did not enhance positive subjective effects in this product category.

Measures of negative PE increased with higher nicotine levels, with the highest nicotine level (12 mg) eliciting significantly greater maximum negative effects compared to NRT gum. These findings suggest that higher nicotine concentrations in Velo Pouch products may result in less favorable subjective experiences, which could indicate a lower potential for AL.

Endpoints assessing UTS relief revealed nuanced patterns. UB cigarette was most effective at alleviating UTS within the first 15 min of use. However, Velo Pouch products, particularly at higher nicotine levels (e.g., 12 mg), provided significantly greater UTS relief than NRT gum and showed no significant differences compared to UB cigarette for longer-term relief (e.g., AUEC_UTS 0–240_, E_min UTS_). These findings highlight the ability of Velo Pouch products to alleviate nicotine cravings effectively, albeit not as rapidly as combustible cigarettes.

Plasma nicotine PK data further support the subjective effects findings. Velo Pouch products demonstrated slower and less pronounced nicotine uptake compared to UB cigarette but greater uptake than NRT gum, except for the highest nicotine level (12 mg), which exhibited a higher overall nicotine exposure comparable to UB cigarette, although this difference was not significant. The T_max_ for Velo Pouch products was significantly longer than for UB cigarette and similar to NRT gum, consistent with buccal absorption mechanisms of these products.

Taken together, the evidence indicates that Velo Pouch products exhibit some level of AL or dependence sustainability driven by their ability to deliver nicotine and relieve UTS. However, this level is lower than that of combustible cigarettes and more closely aligned with NRT gum. The slower nicotine uptake, lower positive PEs, and increased negative PEs at higher nicotine levels further differentiate Velo Pouch products from cigarettes.

These findings suggest that Velo Pouch products, particularly those with lower nicotine levels, may present a reduced potential for abuse compared to cigarettes while maintaining some ability to address nicotine dependence. This supports their potential role in harm reduction strategies, offering smokers a potentially less harmful alternative to combustible cigarettes.

#### Study 2

This study assessed elements of AL of Velo Mini Pouch products (4 mg and 8 mg nicotine levels) compared to UB cigarette (a high-AL comparator) and NRT gum (a low-AL comparator). The assessment was conducted in smokers and dual users of combustible cigarettes and STPs, providing a comprehensive evaluation of the relative AL of Velo Mini Pouch products utilizing subjective and PK endpoints.

The subjective effects data indicate that Velo Mini Pouch products exhibit a moderated AL potential that would support product use or potential for switching from cigarettes or other STPs. Measures of PL were significantly lower for all Velo Mini Pouch products compared to UB cigarette, with most comparisons showing no significant difference from NRT gum. Similarly, positive PE, OPL, and OIUA, were consistently lower for Velo Mini Pouch products than for UB cigarette and largely comparable to or lower than NRT gum.

Negative PEs were generally similar between Velo Mini Pouch products and the comparators. However, one notable exception was the higher AUEC_PEneg 5–240_ observed with Velo Mini Pouch Cool Mint, 8 mg, compared to UB cigarette. These findings suggest that while the Velo Mini Pouch products produce some positive effects, they are attenuated compared to combustible cigarettes, with lower abuse potential indicated by higher nicotine levels eliciting greater negative PE scores.

The ability of Velo Mini Pouch products to relieve UTS was assessed over time. While UB cigarette provided the most rapid UTS relief within the first 15 min of use, Velo Mini Pouch products demonstrated comparable or greater UTS relief over a longer period (4 h) relative to NRT gum. Products with higher nicotine levels (8 mg) were more effective at relieving UTS than lower levels (4 mg), reinforcing the influence of nicotine concentration on AL outcomes.

Plasma nicotine uptake profiles further contextualize the AL potential of Velo Mini Pouch products. Nicotine absorption was slower and less pronounced for Velo Mini Pouch products compared to UB cigarette, with trajectories resembling those of NRT gum. Nicotine uptake increased with higher nicotine levels, with the 8 mg products showing total nicotine uptake comparable to UB cigarette, whereas the 4 mg products demonstrated uptake similar to NRT gum. The T_max_ values for Velo Mini Pouch products were significantly longer than for UB cigarette, reflecting the buccal absorption route and further aligning with NRT gum.

Overall, based on the synthesis of subjective effects and PK data, Velo Mini Pouch products exhibit an intermediate AL profile between UB cigarette and NRT gum. The slower nicotine uptake, together with the reduced positive PEs, and comparable or lower UTS relief relative to NRT gum suggest that these products have a lower abuse potential than combustible cigarettes, particularly at the 4 mg nicotine level. These findings support the potential utility of Velo Mini Pouch products as a lower-risk alternative for smokers seeking harm reduction.

#### Study 3

In Study 3, we examined the potential differences in plasma nicotine uptake and OPL among various Velo Pouch flavors at the same nicotine level to further evaluate AL potential of these products. The findings from this study are consistent with those from Studies one and 2, and provide additional insight into the role of flavor and nicotine content in AL outcomes.

Nicotine PK profiles were generally similar for all Velo Pouch products within the same nicotine level, indicating consistency in nicotine delivery regardless of flavor. The plasma nicotine level for the 10 mg Velo Pouch was higher than that observed for the 8 mg products, as expected with increased nicotine content. Additionally, overall plasma nicotine uptake (AUC_0-240_) and C_max_ were comparable across all flavors of the 8 mg nicotine products, with overlapping 95% confidence intervals. These results indicate that variations in flavor do not considerably influence nicotine absorption or plasma nicotine levels.

OPL was assessed at the end of each test session to capture participants’ subjective preferences for different Velo Pouch products. Ratings of OPL were consistent across all flavors and nicotine levels, suggesting that flavor variations did not substantially alter participants’ liking of the products, supporting the notion that AL potential for these products is driven more by nicotine content than by flavor.

Overall, the findings from Study 3 indicate that nicotine delivery was consistent across flavors at the same nicotine level, and subjective liking remained similar regardless of flavor or nicotine content. By demonstrating consistent PK and subjective outcomes across flavors, this study highlights the potential of Velo Pouch products to maintain a lower AL profile while offering flavor variety, which may support consumer acceptance and adherence as part of harm reduction strategies.

In summary, the assessments of the subjective effect and PK elements that may contribute to the AL of Velo ONPs suggests that these products exhibit lower AL than cigarettes, and comparable to or slightly less AL than NRT gum. Nicotine PK, and therefore AL, were dependent on pouch nicotine content, with increasing levels of nicotine leading to greater nicotine delivery, while subjective effects remained generally similar across products. Oral nicotine pouch flavors (Study 3) and pouch size (Study 1 compared to Study 2), however, did not seem to impact either nicotine PK or subjective effects when these parameters for Velo ONPs are compared to those of cigarettes. Overall, by generating some AL, but to a lesser extent than cigarettes, and by reducing UTS when used, Velo ONPs may offer a viable alternative to cigarettes and support THR at both the individual and population-level.

## Data Availability

The original contributions presented in the study are included in the article/Supplementary Material, further inquiries can be directed to the corresponding author.
