# Supplementary material for: Randomized crossover clinical studies to assess abuse liability and nicotine pharmacokinetics of Velo Oral Nicotine pouches
Source: Front Pharmacol. 2025 Mar 13;16:1547073. doi: 10.3389/fphar.2025.1547073 (PMC11966027; doi:10.3389/fphar.2025.1547073)
Supplement: Supplementary file 1 [file DataSheet1.docx]

Supplementary Material

# Supplementary Figures and Tables

## Supplementary Figure


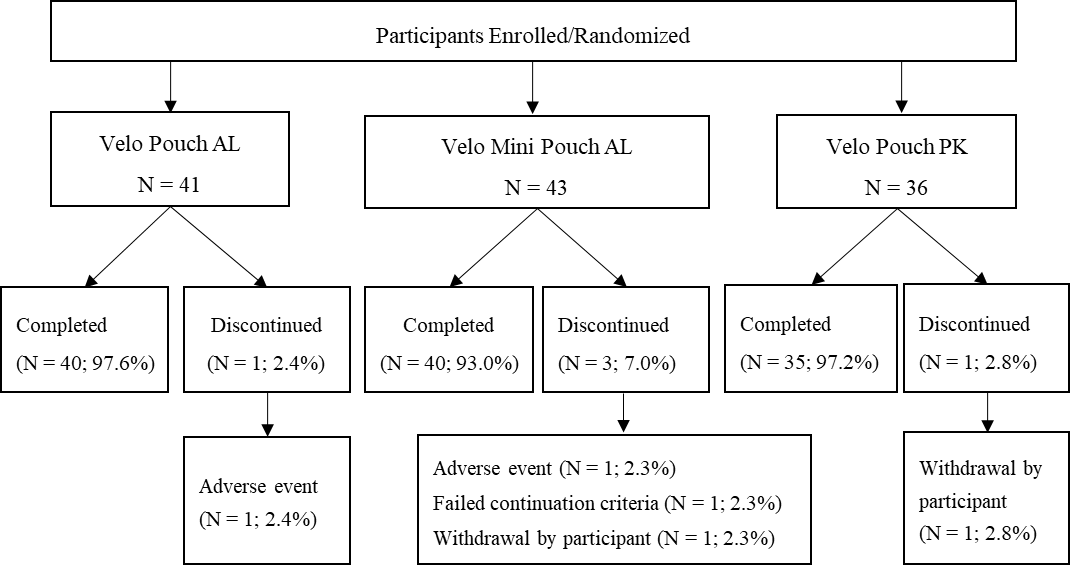


**Supplementary Figure 1.** Disposition of study participants in Velo ONP abuse liability and pharmacokinetic studies. *Abbreviations*: AL, abuse liability; PK, pharmacokinetic; N, number of participants.

## Supplementary Tables

**Supplementary Table 1.** Comparative Analysis of Nicotine Pharmacokinetic Parameters for Velo ONPs Across Studies 1 and 2.

| **Parameter** | **Study 1: Velo Pouch AL** | | | | **Study 2: Velo Mini Pouch AL** | | | |
| --- | --- | --- | --- | --- | --- | --- | --- | --- |
|  | **Comparison** | **DF** | **t-value** | **Probability** | **Comparison** | **DF** | **t-value** | **Probability** |
| AUC_0-15_ (ng x min/mL) | B (Test) vs A (Ref) | 143 | -19.98 | <.0001 | E (Test) vs A (Ref) | 162 | -14.07 | <.0001 |
|  | B (Test) vs N (Ref) | 142 | 7.11 | <.0001 | E (Test) vs N (Ref) | 163 | 1.30 | 0.1952 |
|  | C (Test) vs A (Ref) | 144 | -15.50 | <.0001 | F (Test) vs A (Ref) | 163 | -9.26 | <.0001 |
|  | C (Test) vs N (Ref) | 145 | 11.14 | <.0001 | F (Test) vs N (Ref) | 162 | 5.91 | <.0001 |
|  | D (Test) vs A (Ref) | 143 | -13.22 | <.0001 | G (Test) vs A (Ref) | 164 | -14.44 | <.0001 |
|  | D (Test) vs N (Ref) | 144 | 13.19 | <.0001 | G (Test) vs N (Ref) | 161 | 0.76 | 0.4504 |
|  |  |  |  |  | H (Test) vs A (Ref) | 163 | -10.39 | <.0001 |
|  |  |  |  |  | H (Test) vs N (Ref) | 161 | 4.89 | <.0001 |
| AUC_0-240_ (ng x min/mL) | B (Test) vs A (Ref) | 144 | -9.46 | <.0001 | E (Test) vs A (Ref) | 171 | -1.17 | 0.2445 |
|  | B (Test) vs N (Ref) | 143 | 2.84 | 0.0052 | E (Test) vs N (Ref) | 171 | -0.17 | 0.8625 |
|  | C (Test) vs A (Ref) | 145 | -3.19 | 0.0017 | F (Test) vs A (Ref) | 171 | 4.22 | <.0001 |
|  | C (Test) vs N (Ref) | 145 | 8.96 | <.0001 | F (Test) vs N (Ref) | 171 | 5.27 | <.0001 |
|  | D (Test) vs A (Ref) | 144 | 0.60 | 0.5467 | G (Test) vs A (Ref) | 172 | -2.29 | 0.0234 |
|  | D (Test) vs N (Ref) | 144 | 12.68 | <.0001 | G (Test) vs N (Ref) | 170 | -1.32 | 0.1886 |
|  |  |  |  |  | H (Test) vs A (Ref) | 172 | 3.51 | 0.0006 |
|  |  |  |  |  | H (Test) vs N (Ref) | 170 | 4.57 | <.0001 |
| C_max_ (ng/mL) | B (Test) vs A (Ref) | 145 | -15.21 | <.0001 | E (Test) vs A (Ref) | 173 | -5.30 | <.0001 |
|  | B (Test) vs N (Ref) | 144 | 2.46 | 0.0150 | E (Test) vs N (Ref) | 173 | -0.01 | 0.9904 |
|  | C (Test) vs A (Ref) | 146 | -10.02 | <.0001 | F (Test) vs A (Ref) | 173 | -1.21 | 0.2295 |
|  | C (Test) vs N (Ref) | 146 | 7.43 | <.0001 | F (Test) vs N (Ref) | 173 | 4.06 | <.0001 |
|  | D (Test) vs A (Ref) | 145 | -6.45 | <.0001 | G (Test) vs A (Ref) | 174 | -6.44 | <.0001 |
|  | D (Test) vs N (Ref) | 145 | 10.88 | <.0001 | G (Test) vs N (Ref) | 171 | -1.18 | 0.2401 |
|  |  |  |  |  | H (Test) vs A (Ref) | 174 | -1.64 | 0.1018 |
|  |  |  |  |  | H (Test) vs N (Ref) | 172 | 3.66 | 0.0003 |

Product A: UB cigarette; Product N: Nicorette White Ice Mint polacrilex gum, 4 mg nicotine. Product B, Velo Pouch Cool Mint, 4 mg; Product C, Velo Pouch Cool Mint, 8 mg; Product D, Velo Pouch Cool Mint, 12 mg; Product E, Velo Mini Pouch Cool Mint, 4 mg; Product F, Velo Mini Pouch Cool Mint, 8 mg; Product G, Velo Mini Pouch Modern Traditions, 4 mg; Product H, Velo Mini Pouch Modern Traditions, 8 mg.

*Abbreviations*: AL, abuse liability; AUC, area under the curve; AUC_0-15_, AUC for 0-15 minutes after initiation of product use; AUC_0-240_, AUC for 0-240 minutes after initiation of product use; C_max_, maximum baseline-adjusted plasma nicotine concentration; DF, degrees of freedom; NRT, nicotine replacement therapy; ONPs, oral nicotine pouches; Ref, reference product (UB cigarette); UB, usual brand.

**Supplementary Table 2:** Comparative Analysis of Subjective Effects Parameters for Velo ONPs Across Studies 1 and 2.

| **Parameter** | **Study 1: Velo Pouch AL** | | | | **Study 2: Velo Mini Pouch AL** | | | |
| --- | --- | --- | --- | --- | --- | --- | --- | --- |
|  | **Comparison** | **DF** | **t-value** | **Probability** | **Comparison** | **DF** | **t-value** | **Probability** |
| AUEC_PL 5-240_ | B (Test) vs A (Ref) | 152 | -6.52 | <.0001 | E (Test) vs A (Ref) | 195 | -8.87 | <.0001 |
|  | B (Test) vs N (Ref) | 152 | -0.10 | 0.9176 | E (Test) vs N (Ref) | 195 | -1.64 | 0.1028 |
|  | C (Test) vs A (Ref) | 154 | -6.93 | <.0001 | F (Test) vs A (Ref) | 195 | -8.86 | <.0001 |
|  | C (Test) vs N (Ref) | 154 | -0.50 | 0.6210 | F (Test) vs N (Ref) | 195 | -1.60 | 0.1121 |
|  | D (Test) vs A (Ref) | 152 | -7.00 | <.0001 | G (Test) vs A (Ref) | 195 | -8.68 | <.0001 |
|  | D (Test) vs N (Ref) | 152 | -0.59 | 0.5585 | G (Test) vs N (Ref) | 195 | -1.41 | 0.1594 |
|  |  |  |  |  | H (Test) vs A (Ref) | 195 | -8.45 | <.0001 |
|  |  |  |  |  | H (Test) vs N (Ref) | 195 | -1.18 | 0.2391 |
| E_max PL_ | B (Test) vs A (Ref) | 152 | -6.32 | <.0001 | E (Test) vs A (Ref) | 195 | -7.99 | <.0001 |
|  | B (Test) vs N (Ref) | 152 | -1.06 | 0.2911 | E (Test) vs N (Ref) | 195 | -1.57 | 0.1176 |
|  | C (Test) vs A (Ref) | 153 | -6.28 | <.0001 | F (Test) vs A (Ref) | 195 | -8.32 | <.0001 |
|  | C (Test) vs N (Ref) | 153 | -1.00 | 0.3176 | F (Test) vs N (Ref) | 195 | -1.88 | 0.0623 |
|  | D (Test) vs A (Ref) | 152 | -6.15 | <.0001 | G (Test) vs A (Ref) | 195 | -6.81 | <.0001 |
|  | D (Test) vs N (Ref) | 152 | -0.89 | 0.3767 | G (Test) vs N (Ref) | 195 | -0.35 | 0.7258 |
|  |  |  |  |  | H (Test) vs A (Ref) | 195 | -7.19 | <.0001 |
|  |  |  |  |  | H (Test) vs N (Ref) | 195 | -0.74 | 0.4608 |
| AUEC_UTS 0-15_ | B (Test) vs A (Ref) | 139 | 4.34 | <.0001 | E (Test) vs A (Ref) | 192 | 6.99 | <.0001 |
|  | B (Test) vs N (Ref) | 138 | -0.58 | 0.5620 | E (Test) vs N (Ref) | 192 | 0.26 | 0.7939 |
|  | C (Test) vs A (Ref) | 142 | 2.38 | 0.0189 | F (Test) vs A (Ref) | 193 | 6.96 | <.0001 |
|  | C (Test) vs N (Ref) | 143 | -2.44 | 0.0160 | F (Test) vs N (Ref) | 192 | 0.22 | 0.8278 |
|  | D (Test) vs A (Ref) | 140 | 2.84 | 0.0052 | G (Test) vs A (Ref) | 192 | 7.98 | <.0001 |
|  | D (Test) vs N (Ref) | 140 | -1.94 | 0.0538 | G (Test) vs N (Ref) | 192 | 1.22 | 0.2240 |
|  |  |  |  |  | H (Test) vs A (Ref) | 193 | 7.72 | <.0001 |
|  |  |  |  |  | H (Test) vs N (Ref) | 193 | 1.00 | 0.3181 |
| AUEC_UTS 0-240_ | B (Test) vs A (Ref) | 136 | 1.35 | 0.1792 | E (Test) vs A (Ref) | 189 | 2.23 | 0.0270 |
|  | B (Test) vs N (Ref) | 135 | -0.77 | 0.4446 | E (Test) vs N (Ref) | 189 | 1.04 | 0.3005 |
|  | C (Test) vs A (Ref) | 138 | -0.52 | 0.6055 | F (Test) vs A (Ref) | 190 | 1.37 | 0.1738 |
|  | C (Test) vs N (Ref) | 139 | -2.60 | 0.0104 | F (Test) vs N (Ref) | 189 | 0.17 | 0.8653 |
|  | D (Test) vs A (Ref) | 136 | -0.53 | 0.5983 | G (Test) vs A (Ref) | 189 | 4.33 | <.0001 |
|  | D (Test) vs N (Ref) | 136 | -2.60 | 0.0103 | G (Test) vs N (Ref) | 189 | 3.15 | 0.0019 |
|  |  |  |  |  | H (Test) vs A (Ref) | 190 | 2.76 | 0.0064 |
|  |  |  |  |  | H (Test) vs N (Ref) | 190 | 1.58 | 0.1166 |
| E_min UTS_ | B (Test) vs A (Ref) | 138 | 3.83 | 0.0002 | E (Test) vs A (Ref) | 192 | 7.50 | <.0001 |
|  | B (Test) vs N (Ref) | 137 | -0.77 | 0.4433 | E (Test) vs N (Ref) | 192 | 0.41 | 0.6844 |
|  | C (Test) vs A (Ref) | 140 | 2.29 | 0.0236 | F (Test) vs A (Ref) | 193 | 6.64 | <.0001 |
|  | C (Test) vs N (Ref) | 141 | -2.19 | 0.0305 | F (Test) vs N (Ref) | 193 | -0.46 | 0.6443 |
|  | D (Test) vs A (Ref) | 138 | 1.79 | 0.0751 | G (Test) vs A (Ref) | 192 | 8.68 | <.0001 |
|  | D (Test) vs N (Ref) | 138 | -2.69 | 0.0081 | G (Test) vs N (Ref) | 192 | 1.56 | 0.1208 |
|  |  |  |  |  | H (Test) vs A (Ref) | 194 | 7.86 | <.0001 |
|  |  |  |  |  | H (Test) vs N (Ref) | 193 | 0.79 | 0.4306 |
| AUEC_PEpos 5-240_ | B (Test) vs A (Ref) | 153 | -3.42 | 0.0008 | E (Test) vs A (Ref) | 194 | -5.65 | <.0001 |
|  | B (Test) vs N (Ref) | 153 | 1.67 | 0.0965 | E (Test) vs N (Ref) | 194 | -1.55 | 0.1238 |
|  | C (Test) vs A (Ref) | 153 | -4.23 | <.0001 | F (Test) vs A (Ref) | 194 | -5.85 | <.0001 |
|  | C (Test) vs N (Ref) | 153 | 0.87 | 0.3866 | F (Test) vs N (Ref) | 194 | -1.72 | 0.0864 |
|  | D (Test) vs A (Ref) | 153 | -3.76 | 0.0002 | G (Test) vs A (Ref) | 194 | -6.45 | <.0001 |
|  | D (Test) vs N (Ref) | 153 | 1.32 | 0.1882 | G (Test) vs N (Ref) | 194 | -2.33 | 0.0208 |
|  |  |  |  |  | H (Test) vs A (Ref) | 194 | -5.63 | <.0001 |
|  |  |  |  |  | H (Test) vs N (Ref) | 194 | -1.51 | 0.1321 |
| E_max PEpos_ | B (Test) vs A (Ref) | 152 | -5.37 | <.0001 | E (Test) vs A (Ref) | 195 | -7.17 | <.0001 |
|  | B (Test) vs N (Ref) | 152 | 0.55 | 0.5862 | E (Test) vs N (Ref) | 195 | -0.36 | 0.7217 |
|  | C (Test) vs A (Ref) | 153 | -5.26 | <.0001 | F (Test) vs A (Ref) | 195 | -8.31 | <.0001 |
|  | C (Test) vs N (Ref) | 153 | 0.67 | 0.5021 | F (Test) vs N (Ref) | 195 | -1.48 | 0.1417 |
|  | D (Test) vs A (Ref) | 152 | -5.06 | <.0001 | G (Test) vs A (Ref) | 195 | -8.32 | <.0001 |
|  | D (Test) vs N (Ref) | 152 | 0.86 | 0.3916 | G (Test) vs N (Ref) | 195 | -1.49 | 0.1379 |
|  |  |  |  |  | H (Test) vs A (Ref) | 195 | -8.06 | <.0001 |
|  |  |  |  |  | H (Test) vs N (Ref) | 195 | -1.23 | 0.2204 |
| AUEC_PEneg 5-240_ | B (Test) vs A (Ref) | 152 | -0.01 | 0.9921 | E (Test) vs A (Ref) | 194 | 1.23 | 0.2185 |
|  | B (Test) vs N (Ref) | 152 | 1.06 | 0.2925 | E (Test) vs N (Ref) | 194 | -0.28 | 0.7785 |
|  | C (Test) vs A (Ref) | 153 | 0.75 | 0.4573 | F (Test) vs A (Ref) | 194 | 2.67 | 0.0082 |
|  | C (Test) vs N (Ref) | 153 | 1.81 | 0.0718 | F (Test) vs N (Ref) | 194 | 1.16 | 0.2491 |
|  | D (Test) vs A (Ref) | 152 | 0.90 | 0.3674 | G (Test) vs A (Ref) | 194 | 1.84 | 0.0677 |
|  | D (Test) vs N (Ref) | 152 | 1.97 | 0.0509 | G (Test) vs N (Ref) | 194 | 0.32 | 0.7514 |
|  |  |  |  |  | H (Test) vs A (Ref) | 194 | 0.88 | 0.3777 |
|  |  |  |  |  | H (Test) vs N (Ref) | 194 | -0.64 | 0.5209 |
| E_max PEneg_ | B (Test) vs A (Ref) | 152 | -0.98 | 0.3295 | E (Test) vs A (Ref) | 194 | 0.64 | 0.5251 |
|  | B (Test) vs N (Ref) | 152 | 1.26 | 0.2093 | E (Test) vs N (Ref) | 194 | 0.56 | 0.5775 |
|  | C (Test) vs A (Ref) | 153 | -0.38 | 0.7033 | F (Test) vs A (Ref) | 194 | 1.41 | 0.1589 |
|  | C (Test) vs N (Ref) | 153 | 1.86 | 0.0643 | F (Test) vs N (Ref) | 194 | 1.34 | 0.1811 |
|  | D (Test) vs A (Ref) | 152 | 0.85 | 0.3968 | G (Test) vs A (Ref) | 194 | 1.25 | 0.2120 |
|  | D (Test) vs N (Ref) | 152 | 3.08 | 0.0024 | G (Test) vs N (Ref) | 194 | 1.18 | 0.2398 |
|  |  |  |  |  | H (Test) vs A (Ref) | 194 | 1.28 | 0.2008 |
|  |  |  |  |  | H (Test) vs N (Ref) | 194 | 1.21 | 0.2274 |
| E_overall PL_ | B (Test) vs A (Ref) | 152 | -3.96 | 0.0001 | E (Test) vs A (Ref) | 195 | -8.70 | <.0001 |
|  | B (Test) vs N (Ref) | 152 | 1.59 | 0.1140 | E (Test) vs N (Ref) | 195 | -1.37 | 0.1714 |
|  | C (Test) vs A (Ref) | 153 | -4.90 | <.0001 | F (Test) vs A (Ref) | 195 | -9.01 | <.0001 |
|  | C (Test) vs N (Ref) | 153 | 0.67 | 0.5031 | F (Test) vs N (Ref) | 195 | -1.64 | 0.1017 |
|  | D (Test) vs A (Ref) | 152 | -6.21 | <.0001 | G (Test) vs A (Ref) | 195 | -9.49 | <.0001 |
|  | D (Test) vs N (Ref) | 152 | -0.66 | 0.5100 | G (Test) vs N (Ref) | 195 | -2.13 | 0.0342 |
|  |  |  |  |  | H (Test) vs A (Ref) | 195 | -10.00 | <.0001 |
|  |  |  |  |  | H (Test) vs N (Ref) | 195 | -2.65 | 0.0088 |
| E_overall IUA_ | B (Test) vs A (Ref) | 152 | -6.41 | <.0001 | E (Test) vs A (Ref) | 195 | -10.17 | <.0001 |
|  | B (Test) vs N (Ref) | 152 | 0.60 | 0.5493 | E (Test) vs N (Ref) | 195 | -2.03 | 0.0432 |
|  | C (Test) vs A (Ref) | 154 | -6.91 | <.0001 | F (Test) vs A (Ref) | 195 | -10.05 | <.0001 |
|  | C (Test) vs N (Ref) | 154 | 0.12 | 0.9062 | F (Test) vs N (Ref) | 195 | -1.88 | 0.0619 |
|  | D (Test) vs A (Ref) | 152 | -7.64 | <.0001 | G (Test) vs A (Ref) | 195 | -11.10 | <.0001 |
|  | D (Test) vs N (Ref) | 152 | -0.63 | 0.5273 | G (Test) vs N (Ref) | 195 | -2.93 | 0.0038 |
|  |  |  |  |  | H (Test) vs A (Ref) | 195 | -10.69 | <.0001 |
|  |  |  |  |  | H (Test) vs N (Ref) | 195 | -2.52 | 0.0125 |

Product A, UB cigarette; Product N, Nicorette White Ice Mint polacrilex gum, 4 mg nicotine; Product B, Velo Pouch Cool Mint, 4 mg; Product C, Velo Pouch Cool Mint, 8 mg; Product D, Velo Pouch Cool Mint, 12 mg; Product E, Velo Mini Pouch Cool Mint, 4 mg; Product F, Velo Mini Pouch Cool Mint, 8 mg; Product G: Velo Mini Pouch Modern Traditions, 4 mg; Product H: Velo Mini Pouch Modern Traditions, 8 mg.

*Abbreviations*: AL, abuse liability; AUEC, area-under-the-effect curve; AUEC_PEneg 5-240,_ AUEC for negative PE for 5–240 minutes after the start of product use; AUEC_PEpos 5-240,_ AUEC for positive PE for 5–240 minutes after the start of product use; AUEC_PL5-240,_ AUEC for PL for 5–240 minutes after the start of product use; AUEC_UTS 0-240,_ AUEC for UTS for 0–240 minutes following initiation of product use; AUEC_UTS 0-15,_ AUEC for UTS for 0–15 min following initiation of product use; DF, degrees of freedom; E_max PL_, the maximum PL effect after the start of product use; E_max PEneg,_ maximum positive PE; E_max PEpos_, maximum positive PE; E_min UTS_, minimum UTS; E_overall IUA_ - effect of overall intent to use again at 240 minutes after product use; E_overall PL_- effect of overall PL at 240 minutes after product use; NRT, nicotine replacement therapy; PE, product effects; PL, product liking; Ref, reference product; UB, usual brand; UTS, urge to smoke.

**Supplementary Table 3A.** Summary of severity of reported adverse events and their causal relationship to the study product in Study 1 (Velo Pouch AL)*.

| **Adverse events** | **Velo Pouch** | | | | |
| --- | --- | --- | --- | --- | --- |
|  | **Cool Mint (4 mg) n=40** | **Cool Mint (8 mg) n=41** | **Cool Mint (12 mg) n=40** | **UB cig. n=40** | **NRT gum n=40** |
| No. of participants with AEs | 6 (15.0) | 9 (22.0) | 11 (27.5) | 13 (32.5) | 6 (15.0) |
| Any AE |  |  |  |  |  |
| Causally related to study product | 5 | 6 | 6 | 9 | 3 |
| Possibly related to study product | 5 | 3 | 3 | 3 | 2 |
| Not related to study product | 1 | 3 | 5 | 7 | 1 |
| No. of participants with AE severity |  |  |  |  |  |
| Mild | 6 (15.0) | 9 (22.0) | 11 (27.5) | 13 (32.5) | 5 (12.5) |
| Moderate | 0 | 0 | 0 | 0 | 1 (2.5) |
| Severe | 0 | 0 | 0 | 0 | 0 |
| No. of participants with SAEs | 0 | 0 | 0 | 0 | 0 |
| *Data are reported as number (percentage). *Abbreviations*: AE, adverse event; AL, abuse liability; n, number of observations; SAE, severe AE; UB, usual brand. | | | | | |

**Supplementary Table 3B.** Summary of severity of reported adverse events and their causal relationship to the study product in Study 2 (Velo Mini Pouch AL)*.

| **Adverse event** | **Velo Mini Pouch** | | | | | |
| --- | --- | --- | --- | --- | --- | --- |
|  | **Cool Mint (4 mg)**  **n=40** | **Cool Mint (8 mg)**  **n=41** | **Modern Traditions (4 mg) n=42** | **Modern Traditions (8 mg) n=42** | **UB cig.**  **n=41** | **Nicotine**  **gum**  **n=43** |
| No. of participants with AEs | 1 (2.5) | 3 (7.3) | 3 (7.1) | 0 | 3 (7.3) | 1 (2.3) |
| Any AE |  |  |  |  |  |  |
| Causally related to study product | 0 | 2 | 1 | 0 | 0 | 4 |
| Possibly related to study product | 0 | 2 | 0 | 0 | 0 | 4 |
| Not related to study product | 0 | 0 | 1 | 0 | 0 | 0 |
| No. of participants with AE severity |  |  |  |  |  |  |
| Mild | 1 (2.5) | 3 (7.3) | 3 (7.3) | 0 | 3 (7.3) | 1 (2.3) |
| Moderate | 0 | 0 | 0 | 0 | 0 | 1 (2.3) |
| Severe | 0 | 0 | 0 | 0 | 0 | 0 |
| No. of participants with SAEs | 0 | 0 | 0 | 0 | 0 | 0 |
| *Data are reported as number (percentage). Abbreviations: AE, adverse event; AL, abuse liability; n, number of observations; SAE, severe AE; UB, usual brand. | | | | | | |

**Supplementary Table 3C.** Summary of severity of reported adverse events and their causal relationship to the study product in Study 3 (Velo Pouch PK)*.

| **Adverse event** | **Velo Pouch** | | | | | | |
| --- | --- | --- | --- | --- | --- | --- | --- |
|  | **Berry Frost (8 mg)**  **n=36** | **Cinnamon (8 mg)**  **n=36** | **Cool Mint (8 mg)**  **n=36** | **Smooth (8 mg)**  **n=36** | **Wintergreen (8 mg)**  **n=36** | **Modern Traditions (8 mg) n=36** | **Modern Traditions (10 mg) n=36** |
| No. of participants with AEs | 0 | 2 (5.6) | 0 | 1 (2.8) | 0 | 0 | 0 |
| Any AE |  |  |  |  |  |  |  |
| Causally related to study product | 0 | 1 (2.8) | 0 | 0 | 0 | 0 | 0 |
| Possibly related to study product | 0 | 0 | 0 | 0 | 0 | 0 | 0 |
| Not related to study product | 0 | 1 (2.8) | 0 | 0 | 0 | 0 | 0 |
| No. of participants with AE severity |  |  |  |  |  |  |  |
| Mild | 0 | 2 (5.5) | 0 | 1 (2.8) | 0 | 0 | 0 |
| Moderate | 0 | 0 | 0 | 0 | 0 | 0 | 0 |
| Severe | 0 | 0 | 0 | 0 | 0 | 0 | 0 |
| No. of participants with SAEs | 0 | 0 | 0 | 0 | 0 | 0 | 0 |
| *Data are reported as number (percentage). *Abbreviations*: AE, adverse event; mg, milligram (of nicotine); n, number of participants; PK, pharmacokinetic; SAE, severe AE. | | | | | | | |

**Supplementary Table 4.** Summary of overall reported adverse events in the study populations

| **System organ class preferred term** | **Study 1: Velo Pouch AL**  **n=41** | | **Study 2: Velo Mini Pouch AL**  **n=43** | | **Study 3: Velo Pouch PK**  **n=36** | |
| --- | --- | --- | --- | --- | --- | --- |
|  | **No. of AEs** | **n (%)** | **No. of AEs** | **n (%)** | **No. of AEs** | **n (%)** |
| Any AE | 90 | 29 (70.7) | 18 | 10 (23.3) | 4 | 4 (11.1) |
| **Cardiac disorders** | 0 | 0 | 2 | 2 (4.7) | 0 | 0 |
| Bradycardia | 0 | 0 | 1 | 1 (2.3) | 0 | 0 |
| Tachycardia | 0 | 0 | 1 | 1 (2.3) | 0 | 0 |
| **Ear and labyrinth disorders** | 2 | 1 (2.4) | 0 | 0 | 0 | 0 |
| Tinnitus | 2 | 1 (2.4) | 0 | 0 | 0 | 0 |
| **Gastrointestinal disorders** | 23 | 12 (29.3) | 4 | 3 (7.0) | 1 | 1 (2.8) |
| Abdominal discomfort | 2 | 2 (4.9) | 0 | 0 | 0 | 0 |
| Dyspepsia | 4 | 3 (7.3) | 0 | 0 | 0 | 0 |
| **Gastroesophageal reflux disease** | 1 | 1 (2.4) | 0 | 0 | 0 | 0 |
| Gingival discomfort | 2 | 2 (4.9) | 0 | 0 | 0 | 0 |
| Nausea | 11 | 7 (17.1) | 3 | 2 (4.7) | 0 | 0 |
| Esophageal pain | 1 | 1 (2.4) | 0 | 0 | 0 | 0 |
| Paresthesia oral | 1 | 1 (2.4) | 0 | 0 | 0 | 0 |
| Retching |  |  |  |  | 1 | 1 (2.8) |
| Vomiting | 1 | 1 (2.4) | 1 | 1 (2.3) | 0 | 0 |
| **General disorders and administration site conditions** | 1 | 1 (2.4) | 0 | 0 | 0 | 0 |
| Infusion site hemorrhage | 1 | 1 (2.4) | 0 | 0 | 0 | 0 |
| Hypercalcemia | 0 | 0 | 1 | 1 (2.3) | 0 | 0 |
| Musculoskeletal and connective tissue disorders | 0 | 0 | 1 | 1 (2.3) | 0 | 0 |
| Injury, poisoning and procedural complications | 2 | 1 (2.4) | 1 | 1 (2.3) | 0 | 0 |
| Ligament sprain | 1 | 1 (2.4) | 0 | 0 | 0 | 0 |
| Skin abrasion | 1 | 1 (2.4) | 0 | 0 | 0 | 0 |
| Thermal burn | 0 | 0 | 1 | 1 (2.3) | 0 | 0 |
| Investigations | 2 | 2 (4.9) | 2 | 1 (2.3) | 0 | 0 |
| ALT increased | 0 | 0 | 1 | 1 (2.3) | 0 | 0 |
| AST increased | 0 | 0 | 1 | 1 (2.3) | 0 | 0 |
| Blood pressure systolic increased | 1 | 1 (2.4) | 0 | 0 | 0 | 0 |
| Heart rate increased | 1 | 1 (2.4) | 0 | 0 | 0 | 0 |
| Metabolism and nutrition disorders | 0 | 0 | 1 | 1 (2.3) | 0 | 0 |
| Arthralgia | 0 | 0 | 1 | 1 (2.3) | 0 | 0 |
| Musculoskeletal and connective tissue disorders | 2 | 2 (4.9) | 0 | 0 | 1 | 1 (2.8) |
| Myalgia | 1 | 1 (2.4) | 0 | 0 | 0 | 0 |
| Neck pain | 1 | 1 (2.4) | 0 | 0 | 0 | 0 |
| Pain in extremity | 0 | 0 | 0 | 0 | 1 | 1 (2.8) |
| Nervous system disorders | 35 | 18 (43.9) | 3 | 3 (7.0) | 0 | 0 |
| Dizziness | 12 | 10 (24.4) | 0 | 0 | 0 | 0 |
| Headache | 19 | 14 (34.1) | 2 | 2 (4.7) | 0 | 0 |
| Hyperesthesia | 0 | 0 | 1 | 1 (2.3) | 0 | 0 |
| Paresthesia | 1 | 1 (2.4) | 0 | 0 | 0 | 0 |
| Presyncope | 2 | 2 (4.9) | 0 | 0 | 0 | 0 |
| Somnolence | 1 | 1 (2.4) | 0 | 0 | 0 | 0 |
| Psychiatric disorders | 4 | 1 (2.4) | 0 | 0 | 0 | 0 |
| Euphoric mood | 4 | 1 (2.4) | 0 | 0 | 0 | 0 |
| Reproductive system and breast disorders | 1 | 1 (2.4) | 0 | 0 | 0 | 0 |
| Erection increased | 1 | 1 (2.4) | 0 | 0 | 0 | 0 |
| Respiratory, thoracic, and mediastinal disorder | 7 | 5 (12.2) | 0 | 0 | 0 | 0 |
| Cough | 1 | 1 (2.4) | 0 | 0 | 0 | 0 |
| Hiccups | 4 | 2 (4.9) | 0 | 0 | 0 | 0 |
| Throat irritation | 1 | 1 (2.4) | 0 | 0 | 0 | 0 |
| Tracheal disorder | 1 | 1 (2.4) | 0 | 0 | 0 | 0 |
| Skin and subcutaneous tissue disorders | 3 | 3 (7.3) | 1 | 1 (2.3) | 2 | 2 (5.6) |
| Dermatitis contact | 1 | 1 (2.4) | 0 | 0 | 1 | 1 (2.8) |
| Dry skin | 1 | 1 (2.4) | 0 | 0 | 0 | 0 |
| Ecchymosis | 0 | 0 | 1 | 1 (2.3) | 0 | 0 |
| Hyperhidrosis | 1 | 1 (2.4) | 0 | 0 | 0 | 0 |
| Skin abrasion | 0 | 0 | 0 | 0 | 1 | 1 (2.8) |
| Vascular disorder | 8 | 7 (17.1) | 3 | 2 (4.7) | 0 | 0 |
| Flushing | 0 | 0 | 1 | 1 (2.3) | 0 | 0 |
| Hypertension | 8 | 7 (17.1) | 1 | 1 (2.3) | 0 | 0 |
| Hypotension | 0 | 0 | 1 | 1 (2.3) | 0 | 0 |
| Abbreviations: AE, adverse event; AL, abuse liability; n, number of observations; PK, pharmacokinetic | | | | | | |
